# ASF1B promotes erythropoiesis by regulating the establishment and enrichment of H3.3 nucleosomes

**DOI:** 10.1093/nar/gkag447

**Published:** 2026-05-08

**Authors:** Jinlei Liu, Xuemei Song, Lecong Zhou, Yangu Zhao, Juhyun Kim, Jun Li, Ann Dean, Xiang Guo

**Affiliations:** Department of Hematology, Sichuan Provincial People’s Hospital, School of Medicine, University of Electronic Science and Technology of China, Chengdu 610072, China; Department of Hematology, Sichuan Provincial People’s Hospital, School of Medicine, University of Electronic Science and Technology of China, Chengdu 610072, China; Laboratory of Cellular and Developmental Biology, National Institute of Diabetes and Digestive and Kidney Diseases, National Institutes of Health, Bethesda, MD 20892, United States; Laboratory of Cellular and Developmental Biology, National Institute of Diabetes and Digestive and Kidney Diseases, National Institutes of Health, Bethesda, MD 20892, United States; Laboratory of Cellular and Developmental Biology, National Institute of Diabetes and Digestive and Kidney Diseases, National Institutes of Health, Bethesda, MD 20892, United States; Department of Otolaryngology-Head & Neck Surgery, West China Hospital, Sichuan University, Chengdu 610041, China; Laboratory of Cellular and Developmental Biology, National Institute of Diabetes and Digestive and Kidney Diseases, National Institutes of Health, Bethesda, MD 20892, United States; Department of Hematology, Sichuan Provincial People’s Hospital, School of Medicine, University of Electronic Science and Technology of China, Chengdu 610072, China

## Abstract

Histone chaperone ASF1B is largely known to be functionally correlated with cell proliferation and cell cycle. We found that the expression of ASF1B was abundant together with histone variant H3.3 during in mouse fetal liver cells. However, the mechanism underlying this coordination is still unclear. HIRA, a H3.3 specific chaperone, was dispensable in fetal hematopoiesis. In contrast, we found that ASF1B predominantly regulated H3.3 encoding genes and erythroid genes, whereas ASF1A served a compensatory function. Notably, ASF1B occupied >70% of H3.3 nucleosomes and determined H3.3 enrichment at erythroid gene promoters and enhancers. However, loss of ASF1B de-repressed the expression of embryonic/fetal globin genes by altering enrichment of H3.3 and erythroid transcription factors as well as chromatin accessibility. The regulatory pathway of ASF1B in H3.3 enrichment involved the recruitment of chromatin remodeler BRG1 and accumulation of H3K27ac in active chromatin. In summary, ASF1B plays a crucial role in enrichment of H3.3 nucleosomes and establishment of the chromatin environment to affect erythroid gene expression, highlighting the therapeutic potential of ASF1B in targeting erythrocyte disorders, such as β-globin hemoglobinopathies.

## Graphical Abstract

**Graphical Abstract:**
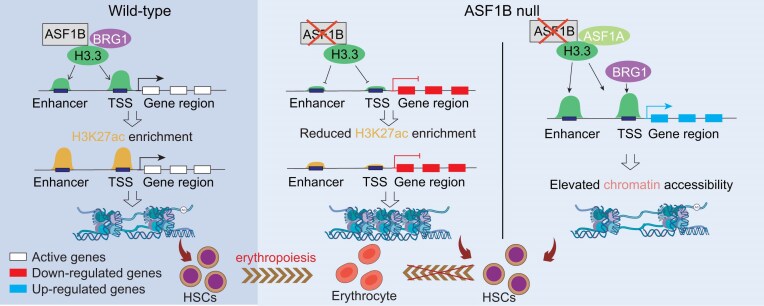

## Introduction

The nucleosome, the fundamental element of chromatin, is an octamer comprising two copies of each of the four core histone proteins (H2A, H2B, H3, and H4), which is wrapped by about 146 bp of DNA [1]. H3.1, H3.2, and H3.3 are the major isoforms of the histone H3 family in mammals [2, 3]. The deposition of H3.1 and H3.2 into chromatin is mediated by chromatin assembly factor 1 (CAF-1) in a DNA replication-coupled (RC) nucleosome assembly manner [4, 5]. H3.3 is incorporated into nucleosomes in a DNA replication-independent (RI) manner by the histone repression A factor (HIRA) [5, 6]. Anti-silencing function 1 (ASF1), a histone H3–H4 chaperone, also participates in H3.1/H3.3 nucleosome assembly. The highly conserved N-terminus of ASF1 serves as a binding interface with H3.1/H3.2–H4 histones or H3.3–H4 histones [7]. Moreover, ASF1 transmits newly synthesized soluble H3/H4 histones to CAF-1 and HIRA in the RC and RI nucleosome assembly pathways, respectively [8].

There are two mammalian paralogs of ASF1, ASF1A and ASF1B, which share 70% sequence identity but exhibit distinct expression patterns and functional roles [9]. ASF1A is widespread in different cell types and is mainly involved in DNA repair and cell senescence [8, 10]. ASF1B is highly expressed in multiple cancer cells where it primarily contributes to cell proliferation [8, 10–15]. Both ASF1A and ASF1B participate in chromatin assembly but function differently. ASF1A complexes contain HIRA for RI nucleosome assembly of H3.3. ASF1B is not involved in these complexes even though it can directly interact with the B-domain of HIRA [5, 9, 16]. ASF1B promotes pancreatic β-cell proliferation by recruiting H3.3, providing an example of functional correlation [12]. However, the regulatory networks of ASF1A, ASF1B, and HIRA in H3.3 nucleosomes remain to be further investigated.

Histone H3.3, encoded by *H3f3a* and *H3f3b*, was originally identified as a component of active chromatin, particularly enriched in actively transcribed gene bodies and promoters [17–19]. The replacement of H3 by H3.3 in intergenic regions suggests a potential role for H3.3 at regulatory elements such as active enhancers. H3.3 nucleosomes are found in conjunction with the histone variant H2A.Z at active regulatory sites such as the β-globin locus in erythroid cells [20, 21]. Moreover, H3.3 is essential for hematopoietic stem cell (HSCs) homeostasis and stemness through modulating chromatin at bivalent genes marked by H3K4me3 and H3K27me3 in adult mice [22]. Despite the significance of H3.3 function in hematopoiesis, the H3.3 chaperone HIRA, while necessary for the self-renewal and survival of adult HSCs, is dispensable for fetal hematopoiesis [23, 24]. This observation raises the question which pathway is involved in H3.3 deposition for gene regulation during fetal erythropoiesis.

**Figure 1.**
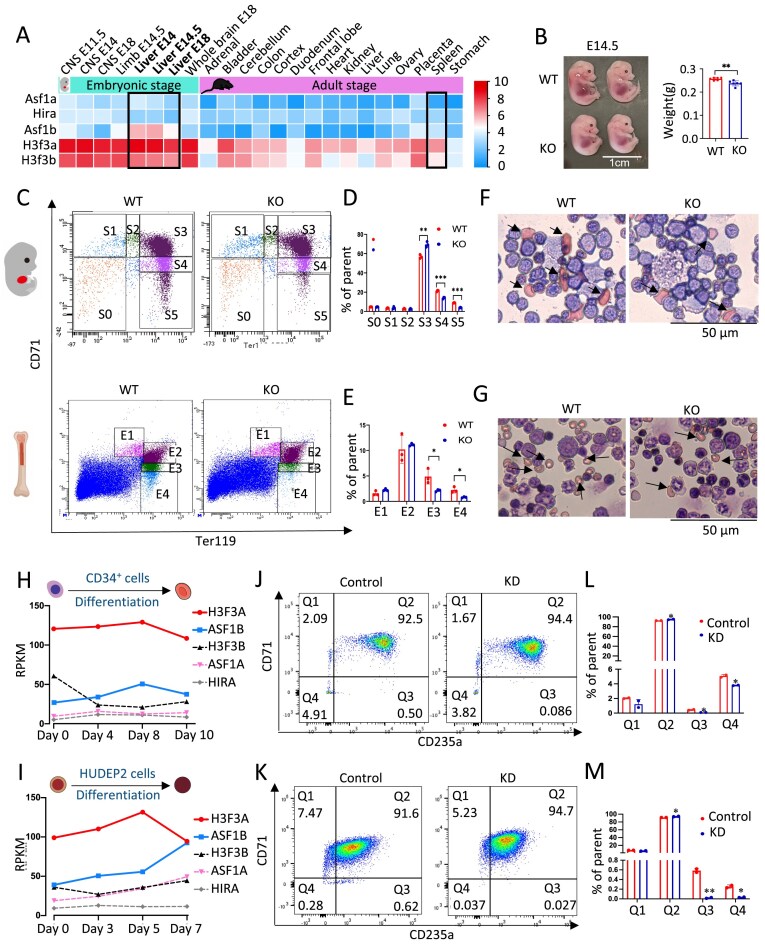
High levels of Asf1b gene expression were important for normal mouse and human erythropoiesis. (**A**) Normalized expression level of *Asf1a, Asf1b, Hira, H3f3a*, and *H3f3b* gene in multiple mouse tissues (log_2_RPKM (Reads Per Kilobase per Million mapped reads), data adapted from Encode PRJNA66167). (**B**) E14.5 embryos of *Asf1b ^−/−^* mice are smaller in size compared to control littermates; scale bar: 1 cm. (**C**) Flow cytometry with Ter119 (glycophorin A, GYPA) and CD71 (transferrin receptor) staining to separate maturational stages S0–S5 E14.5 liver cells (upper) and E1–E4 bone marrow (BM) cells (bottom) of *Asf1b ^−/−^* and WT mice. (**D** and **E**) Quantification of cell numbers of S0–S5 (D) and E1–E4 (E) population in panel (C). (**F** and **G**) Wright–Giemsa staining showing less mature red cells of *Asf1b ^−/−^* E14.5 liver cells (F) and BM cells (G), which are compared to controls. (**H** and **I**) Normalized expression level (RPKM) of *H3F3A, ASF1B, H3F3B, ASF1A*, and *HIRA* gene during the differentiation of CD34^+^ cells (day 0, 4, 8, and 10) and HUDEP2 cells (day 0, 3, 5, and 7) (data adapted from NCBI SRA database SRP338105). (**J** and **K**) Flow cytometry with CD235a and CD71 staining with KD of *ASF1B* in CD34^+^ cells (J), in HUDEP2 cells (K), and the control cells. (**L** and **M**) Quantification of cell numbers in panels (J) and (K); bar: 50 μm. black arrows indicate the mature red cells in panels (F) and (G). Error bars in panels (B), (D), (E), (L), and (M) represent SD. Representative of *N* = 6, B; *N* = 3, D and E; *N* = 2, L and M independent biological replicates. *P < 0.05, **P < 0.01, ***P < 0.001 by two-tailed Student’s *t*-test.

ASF1B is highly abundant in murine fetal liver cells (E14–E14.5), which coincides with high expression of *H3f3a* and *H3f3b*, encoding H3.3 (Fig. 1A). Here, we investigated the role of ASF1B in H3.3 enrichment and chromatin remodeling during fetal erythropoiesis. Compared to H3.3 histone chaperone HIRA, ASF1B predominately determined the transcription, protein level and global distribution of H3.3, whereas the related family number ASF1A served a compensatory function. ASF1B recruited the SWI/SNF complex ATPase BRG1 as a new pathway to accumulation of H3.3 and establishment of active chromatin. Notably, loss of ASF1B de-repressed the expression of embryonic/fetal globin genes by mediating H3.3 enrichment and chromatin accessibility. Overall, ASF1B emerges as an important factor for erythropoiesis through regulating the establishment of H3.3 nucleosomes at erythroid genes.

## Materials and methods

### Mice

An HA tag was inserted into the mouse *Asf1b* gene by CRISPR–Cas9 technology Germplasm of ASF1B KO mice was obtained from MMRRC (MGI:5907638) and mice were generated by *in vitro* fertilization (IVF). *Asf1b^−/-^* mice are viable and fertile but have a reduced reproductive capacity (Messiaen 2016 Reproduction). *Brg1fl/fl* mouse lines were a kind gift of Dr. Trevor Archer, NIEHS, NIH [25]. *Brg1fl/fl* homozygous mice were bred with Vav-iCre mice (Jackson Laboratories) to obtain *fl/+* and *fl/+ Vav-iCre* mice. Stud males were bred to these heterozygous animals. The genotypes of *fl/fl* Vav-iCre embryos and control littermates was checked by western blot analysis. Mouse protocols were approved by the NIDDK Animal Care and Use Committee in accordance with AALAC specifications. For details of mouse strains, breeding, and gene editing, see Supplementary Materials and methods.

### Fluorescence-activated cell sorting and Wright–Giemsa staining

Fetal livers and bone marrow were manually dissociated to single cell suspensions. Wright–Giemsa staining was performed according to the manufacturer’s protocol (WG16, Sigma–Aldrich). Fetal liver [26, 27] and bone marrow cells [28] were separated and purified into multiple events through BD FACS Aria III Cell Sorter. HUDEP2 and CD34^+^ cells were stained with CD71 and CD235a antibodies and analyzed on a BD FACS Canto II. For antibodies, see Supplementary Table S1.

### Cell culture

HUDEP-2 cells were maintained in the medium containing serum-free expansion medium (SFEM) supplemented with human SCF (50 ng/ml), dexamethasone (0.4 μg/ml), doxycycline (1 μg/ml), erythropoietin (3 units/ml), and 1% penicillin/streptomycin. HUDEP2 cells were induced in Iscove’s Modified Dulbecco’s medium (IMDM) containing 5% human AB plasma, 1% L-glutamine, holo-transferrin (500 μg/ml), human insulin (10 μg/ml), erythropoietin (3 units/ml), human SCF (100 ng/ml), heparin (3 units/ml), doxycycline (1 μg/ml), and 2% penicillin/streptomycin as previously described [29]. At 4 days post-induction, the medium was replaced with the same formulation without human SCF. CD34+ cells (Lonza, cat. #2C-101) were cultured as previously described [30]. Expansion phase: cells were cultured in SFEM supplemented with human SCF (50 ng/ml), erythropoietin (2 units/ml), dexamethasone (10^−6^ M), β-estradiol (10^−6^ M), IL-3 (1 ng/ml), and 1% penicillin/streptomycin. Differentiation phase: Medium was changed to SFEM with erythropoietin (2 units/ml), holo-transferrin (1 mg/ml), and 1% penicillin/streptomycin. K562 and MEL cells were cultured in RPMI1640 medium supplemented with 10% fetal bovine serum [31]. K562 cells were induced with 30 μM hemin for 2 days. MEL cells were induced with 2% Dimethyl sulfoxide (DMSO) for 5 days.

### RNAi and lenti-virus production

Lentivirus was produced by transfecting HEK293T cells with pLKO.1, pMD2.G, and psPAX2 in a ratio of 4:1:3 using Lipo8000^™^ Transfection Reagent (Beyotime, C0533), following the Addgene protocol. Crude viral supernatant was harvested, filtered through a 0.45 μm membrane, concentrated by Lenti-X^TM^ Concentrator (Cat# 631232), and subsequently was incubated with HUDEP2 cells in the presence of 4 μg/ml polybrene for 7 days. CD34^+^ cells were electroporated using the 4D-Nucleofector^®^ X Unit (Lonza, AAF-1003X). 1–5 × 10⁶ CD34^+^cells were resuspended in 100 μl of P3 Primary Cell 4D-Nucleofector^™^ X Solution, transferred to a 100-μl Nucleocuvette^™^ (Lonza, V4XP-3024) containing 1–5 μg of plasmid DNA, and electroporated using program EO-100. For shRNA sequences, see Supplementary Table S2.

### RNA sequencing

RNA of E14.5 liver and bone marrow sorted cells was isolated by PicoPure^™^ RNA Isolation Kit (Thermo Fisher, KIT0204). RNAs (25–100 ng) of fetal liver S0–S5 population and bone marrow E1–E4 population were used for RNA-seq library preparation following manufacturer’s protocol (Illumina 20040534). MEL and HUDEP2 cell RNA was isolated by TRIZOL reagent (Abclonal, rk30129). Hundred nanograms of RNAs of MEL and HUDEP2 cells were prepared for RNA-seq library following Illumina’s protocol (20040534). CD34^+^ cell RNA was isolated by ABclonal kit (RK20310). One nanogram of CD34 + cell RNA was used for RNA-seq library preparation following manufacturer’s protocol (ABclonal RK20237).

### CRISPR–Cas9 gene editing

The sgRNAs targeting *Asf1a, Asf1b*, and *Hira* were designed using the CHOP tool available at https://chopchop.cbu.uib.no. The single guide RNAs (sgRNAs) were electroporated into MEL cells using the Neon^™^ Transfection System 100 μl Kit (MPK1096E), following the manufacturer’s protocol. Single-cell clones were isolated by fluorescence-activated cell sorting (FACS) based on GFP expression 48 h post-electroporation. For sgRNA sequences, see Supplementary Table S2.

### Western blotting and co-immunoprecipitation

Nuclear protein extraction and western blotting were performed as previously described [32, 33]. Cells were lysed in a hypotonic buffer 20 mM Tris–HCl, 1 mM NaCl, 3 mM MgCl_2_ containing 1% protease inhibitor cocktail for 15 min on ice. NP40 was then added, and the homogenate was centrifuged for 10 min at 4°C. The nuclear pellet was resuspended in cell lysis buffer (Thermo 87787) and maintained under constant agitation for 30 min at 4°C. Subsequently, the lysate was centrifuged at 4°C for 10 min to extract proteins. For western blot analysis, proteins were separated by SDS–PAGE and transferred onto a Polyvinylidene Difluoride (PVDF) membrane. The membrane was blocked with 5% skim milk for 1 h and then incubated with primary antibodies overnight at 4°C. Following this, the membrane was incubated with secondary antibodies for 1 h at room temperature. Protein signals were detected using a chemiluminescence system. For co-immunoprecipitation, the cell lysate was incubated with primary antibodies and rotated overnight at 4°C, followed by a 2-h incubation with protein A-conjugated beads (Invitrogen, 10006D). For antibodies, see Supplementary Table S1.

### RT-qPCR

The cDNA was synthesized following the manufacturer’s instructions (ThermoFisher Scientific 18080-400 for fetal liver cells; Abclonal RK20433 for CD34^+^, HUDEP2, and MEL cells). Quantitative PCR was performed using iTaq Universal SYBR Green Super Mix (BioRad, 172-5120). For PCR primer sequences, see Supplementary Table S2.

### Chromatin immunoprecipitation-qPCR and sequencing (ChIP-qPCR and ChIP-seq)

ChIP-qPCR and ChIP-seq were performed as previously described [34]. A total of 30 × 10^6^ cells were crosslinked in PBS containing 0.6% formaldehyde and 0.6% paraformaldehyde for 10 min at room temperature and quenched with 0.125 M glycine. Cells were lysed in the HEPES lysis buffer (5 mM HEPES, 150 mM NaCl, 1 mM EDTA, 10% glycerol, 0.5% NP-40, 0.25% Triton X-100, and 1 × protease inhibitor) for 15 min at 4°C. Following centrifugation, chromatin was resuspended in sonication buffer (10 mM Tris, pH 8.0, 0.1% SDS, 2 mM EDTA, and 1 × proteinase inhibitors) and sonicated to an average fragment size of 300 bp using a Bioruptor (Diagenode). For each ChIP, sonicated chromatin from 5 × 10^6^ cells was incubated with 3 µg of antibody overnight at 4°C, followed by a 2-h incubation with Protein A Dynabeads (Invitrogen, 88846) at 4°C. Beads were washed sequentially with low-salt (20 mM Tris–HCl, pH 8.0, 140 mM NaCl, 2 mM EDTA, 0.8% NP40, and 0.1% SDS) two times, high-salt (20 mM Tris–HCl, pH 8.1, 500 mM NaCl, 2 mM EDTA, 0.8% NP40, and 0.04% SDS) two times, and final LiCl washing buffer (10 mM Tris–HCl, 0.25 M LiCl, 1 mM EDTA, 1% NP40, and 1% deoxycholate, pH 8.0). DNA was reverse crosslinked at 65°C overnight and purified using a PCI (phenol:chloroform:isoamyl alcohol, 25:24:1) extraction followed by isopropanol precipitation. Libraries were prepared using the ThruPLEX-DNA-seq Kit (Takara, R400674). Real-time qPCR was performed using the iTaq Universal SYBR Green Super Mix (Bio-Rad, 172-5120). The comparative CT method was applied to calculate the relative enrichment of sequences of interest over input. Antibody details are listed in Supplementary Table S2. For ChIP-qPCR primer and antibodies, see Supplementary Tables 1 and 2, respectively.

### ATAC-seq

E14.5 fetal liver cells (∼50 000) were treated according to the Omni-ATAC-seq protocol (https://www.med.upenn.edu/kaestnerlab/protocols.html) from the Kaestner Lab. E14.5 fetal liver cells (∼50 000) were used for each library. TDE1 Tagment DNA Enzyme (Illumina; catalog no. 15027865) was added to the transposition mixture and incubated for 1 h at 37°C with 1000 rpm shaking. Reactions were clarified with a MinElute Reaction Cleanup Kit (Qiagen, catalog 28204) Libraries were amplified with NEBNext High Fidelity 2 × PCR Master Mix (NEB, M0541S), clarified and size selected with AMPure XP magnetic beads (Beckman Coulter; catalog no. A63880). Library quality was validated with an Agilent Bioanalyzer 2100.

### Data analysis and data sharing

For complete data analysis, see Supplementary Materials and methods. RNA-seq, ChIP-seq, and ATAC-seq data are available at GEO under accession numbers GSE215076, GSE214528 and GSE285838.

## Results

### ASF1B is highly enriched in mouse fetal liver cells and is important for mouse and human erythroid differentiation

ASF1A delivered H3.3–H4 dimers on the HIRA complex and is essential for H3.3 deposition in human and Drosophila cells [35, 36]. However, in the fetal liver (E14 and E14.5), among 23 mouse tissues examined, *Asf1b* exhibited high expression levels compared to *Asf1a* and *Hira*, along with H3.3 encoding genes *H3f3a* and *H3f3b* (Fig. 1A). The liver is the primary site of hematopoiesis in the mouse embryo. *Asf1b* KO mouse E14.5 embryos and fetal livers were smaller compared to controls (Fig. 1B and Supplementary Fig. S1A). Loss of *Asf1b* significantly reduced the numbers of multi-potent progenitors (MPPs) and megakaryocyte-erythroid progenitors (MEPs) revealed by systematic examination of FACS (Supplementary Fig. S1B and C), underscoring a potential role of ASF1B in erythropoiesis. But this loss did not affect long-term hematopoietic stem cells (LT-HSCs), short-term hematopoietic stem cells (ST-HSCs), common myeloid progenitors (CMPs), or granulocyte progenitors (GMPs), supporting a unique regulatory role for ASF1B in erythropoiesis (Supplementary Fig. S1C).

Consistently, erythroid maturation was impaired from E12.5 to E17.5 after deletion of *Asf1b*, as revealed by FACS analysis and staining with erythroid cell surface markers CD71 and Ter119. Mature cell population S4/5 decreased in *Asf1b ^−/−^* fetal livers, while less mature cell population S3 increased compared to controls (Fig. 1C and D, and Supplementary Fig. S1D). Although the expression levels of *Asf1a, Asf1b*, and *Hira* declined significantly from fetal to adult development, deletion of *Asf1b* in adult bone marrow resulted in a reduction of mature cell population E3/E4 (Fig. 1C and E, and Supplementary Fig. S1E). Furthermore, Wright–Giemsa staining of E14.5 liver and adult bone marrow confirmed impaired maturation, with reduced numbers of mature red cells in *Asf1b* KO samples (Fig. 1F and G, and Supplementary Fig. S1F and G).

In human umbilical cord CD34^+^ hematopoietic stem/progenitors (HSPCs) and immortalized HUDEP2 progenitor cells, ASF1B and H3F3A also display high-level expression during erythroid differentiation (Fig. 1H and I). Depletion of ASF1B using shRNA resulted in elevated numbers of CD71 and CD235a double positive cells and reduced mature cell populations, similar to what we detected in mouse tissue (Fig. 1J–M and Supplementary Fig. S2). As expected, the effect H3.3 depletion on erythroid differentiation was similar to depletion of ASF1B in HUDEP2 cells (Supplementary Fig. S3). Collectively, these results implicate *Asf1b* as a cell-intrinsic regulator of H3.3 during mammalian erythropoiesis.

### ASF1B specifically regulates the expression of genes function in the assembly of H3.3 chromatin during erythropoiesis

**Figure 2.**
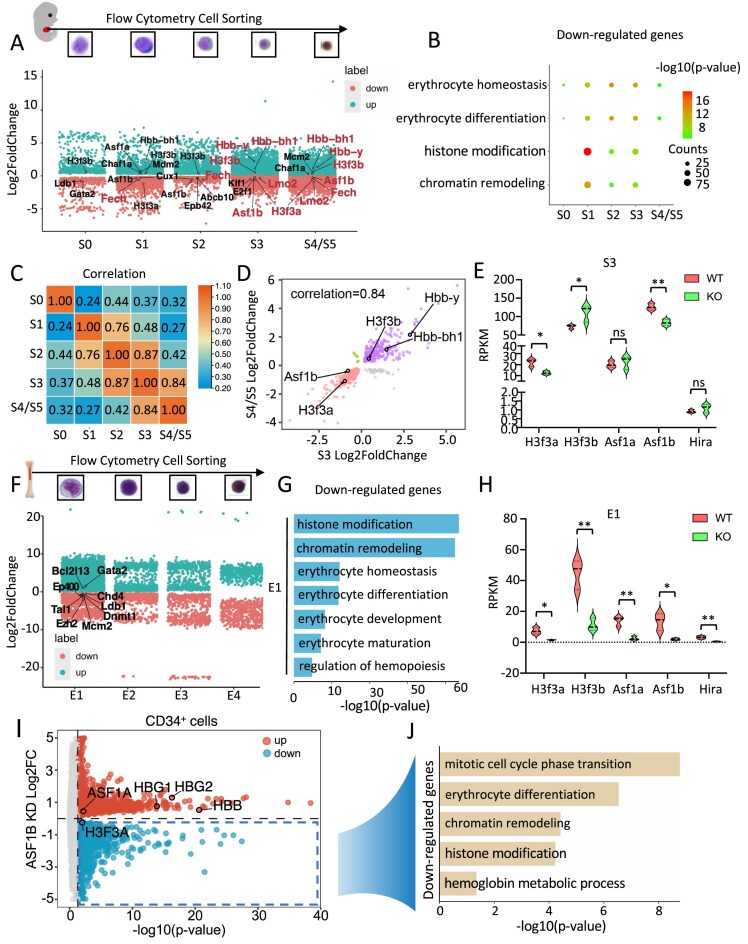
Loss of ASF1B resulted in transcriptional alteration of genes associated with H3.3 chromatin assembly. (**A**) Volcano plot showing the DEGs across five clusters (S0–S5 population) in *Asf1b^−/−^* compared to WT E14.5 liver cells. Genes with significant changed expression in S3–S5 population are labeled in red. *P*-value < 0.05. (**B**) Gene ontology terms enriched among genes significantly downregulated upon *Asf1b* KO in S0–S5 population cells. (**C**) Correlation heatmap of DEGs in *Asf1b^−/−^* compared to WT E14.5 liver cells across S0–S5 population. (**D**) L2fc–L2fc plot comparing two sets of dysregulated genes between S3 and S4/S5 population upon *Asf1b* KO. (**E**) *H3f3a, H3f3b, Asf1a, Asf1b*, and *Hira* gene expression level in RNA-seq of S3 population in WT and *Asf1b^−/-^* cells. (**F**) Volcano plot showing the DEGs across four clusters (E1–E4 population) in *Asf1b^−/−^* compared to WT bone marrow cells. (**G**) Gene ontology terms enriched among genes significantly downregulated upon *Asf1b* KO in E1 population cells. (**H**) *H3f3a, H3f3b, Asf1a, Asf1b*, and *Hira* gene expression level in RNA-seq of E1 population in WT and *Asf1b^−/-^* cells. (**I**) Volcano plot showing DEGs in *ASF1B KD* CD34^+^ cells compared to control. (**J**) Gene ontology terms enriched among genes significantly downregulated upon *ASF1B* KO in CD34^+^ cells. Error bars in panels (E) and (H) represent SD. Representative of *N* = 3, E and H independent biological replicates. *P < 0.05, **P < 0.01 by two-tailed Student’s *t*-test.

To clarify the significance of high expression of genes encoding ASF1B and H3.3, we examined the erythroid transcriptome regulated by Asf1b through RNA sequencing (RNA-seq) in fetal liver S0–S5 populations (Fig. 2A and Supplementary Fig. S4A). The S4 and S5 populations were combined due to the low cell number collected by FACS. In general, Gene Ontology (GO) analysis indicated that dysregulated genes after *Asf1b* deletion were highly enriched in pathways related to histone modification, chromatin remodeling, erythroid differentiation, cell cycle phase transition and intrinsic apoptotic signaling (Fig. 2B, and Supplementary Fig. S4B and C). Especially, key genes involved in H3.3 chromatin assembly were dysregulated, such as CAF-1 complex components *Chaf1a* and *Chaf1b*, and HIRA complex component *Ubn1* and *Asf1a*. The expression of H3.3 encoding gene *H3f3a* was impaired while *H3f3b* gene expression increased, potentially compensating for this loss after *Asf1b* KO during erythrocyte development (Fig. 2A).

In total, 1003–4723 differentially expressed genes (DEGs) were detected in fetal liver S0–S5 ASF1B KO populations (Supplementary Fig. S4D–H). Erythroid-related genes revealed by previous studies [37–39] represented 15%–20% of DEGs during fetal development (Supplementary Fig. S4D–H). Although genes were both upregulated and downregulated upon ASF1B loss, there was a preponderance of downregulated DEGs, especially at the most mature stages S4 and S5, suggesting that ASF1B anti-silencing effect is important for maintaining erythroid gene expression at the fetal stage (Supplementary Fig. S4I). For examples, *Asf1b* loss resulted in developmental downregulation of erythroid genes *Fech* and *Lmo2*. In contrast, the expression of embryonic globin genes *Hbb-bh1* and *Hbb-y* were significantly increased especially in the mature erythroid cell S3–S5 populations (Fig. 2A). DEGs showed a strong correlation in terminal mature cell populations S3 and S4/5, with decreased expression of *H3f3a*/*Asf1b* and increased expression of *H3f3b*/*Asf1a* (Fig. 2C–E and Supplementary Fig. S4J). However, only 18 down-regulated genes and 12 up-regulated genes were shared across development (Supplementary Fig. S4K–N).

In contrast to fetal erythropoiesis, loss of ASF1B primarily dysregulated genes in the E1 population of adult bone marrow cells that were associated with pathways of histone modification, chromatin remodeling, erythroid differentiation, cell cycle, and cell apoptosis (Fig. 2F and G, and Supplementary Fig. S5A–G). Similar to the fetal stage, erythroid-related genes exhibited 15%–20% of DEGs in bone marrow cells (Supplementary Fig. S5B–E). Few genes were dysregulated across E1–E4 populations by *Asf1b* loss (Supplementary Fig. S5H–K). But genes involved in H3.3 nucleosome assembly, such as *H3f3a* and *H3f3b*, histone chaperone *Asf1a* and *Atrx* were significantly altered upon deletion of *Asf1b* (Fig. 2H).

Notably, the regulatory role of ASF1B in H3.3 chromatin assembly, embryonic/fetal globin gene expression, and cell cycle was highly conserved in human CD34^+^ cells (Figs 1 and 2I–J). In summary, our results indicate that ASF1B regulates the transcription of genes encoding H3.3 and genes that function in the pathway of H3.3 chromatin assembly during mammalian erythrocyte development.

### ASF1A can compensate for ASF1B in regulation of H3.3 and erythroid genes

**Figure 3.**
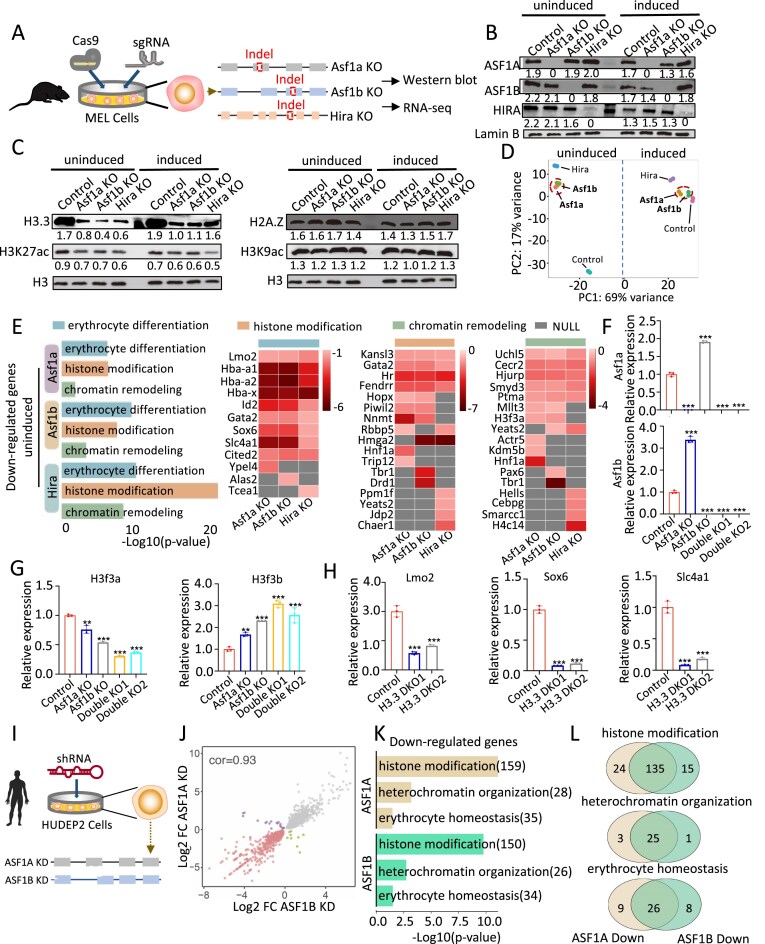
The similarity of regulatory function between ASF1A and ASF1B in H3.3 encoding genes and erythroid genes. (**A**) Schematic diagram of sgRNA-mediated KO of *Asf1a, Asf1b*, and *Hira* gene in MEL cells. (**B** and **C**) Western blotting showing the protein level of ASF1A, ASF1B, HIRA, H3.3, H3K27ac, H2A.Z and H3K9ac in *Asf1a, Asf1b, Hira* KO, and control MEL cells. Relative grayscale values normalized to Lamin B (B) and histone H3 (C) were shown below the bands. (**D**) PCA analysis of RNA-seq in *Asf1a, Asf1b*, and *Hira* KO compared to control MEL cells in uninduced (left panel) and induced (right panel) conditions. (**E**) GO terms (left) enriched among genes significantly downregulated upon *Asf1a, Asf1b*, and *Hira* KO in uninduced MEL cells. Blue represents erythrocyte differentiation pathways, yellow represents histone modification pathways, and green represents chromatin remodeling pathways. Each pathway shows the top ten DEGs in the heatmaps on the right. Gray represents genes showing no significance or not including in the top ten DEGs. (**F** and **G**) RT-PCR showing *Asf1a, Asf1b, H3f3a*, and *H3f3b* gene expression in *Asf1a* KO, *Asf1b* KO, double KO, and control MEL cells. (**H**) RT-PCR showing *Lmo2, Sox6*, and *Slc4a1* gene expression in H3.3 double KO and control MEL cells. *n* = 3 biological replicates. *P < 0.05, **P < 0.01, ***P < 0.001 by two-tailed Student’s *t*-test. (**I**) Schematic diagram of shRNA-mediated KD of *ASF1A* and *ASF1B* gene in human HUDEP2 cells. (**J**) L2fc plot comparing dysregulated genes upon *ASF1A* and *ASF1B* KD in HUDEP2 cells. (**K**) GO terms enriched among genes significantly downregulated upon *ASF1A* and *ASF1B* KD in HUDEP2 cells. (**L**) Venn diagram showing the number of overlapped DEGs enriched in pathways related to histone modification (top), heterochromatin organization (middle) and erythrocyte homeostasis (bottom) upon *ASF1A* KD and *ASF1B* KD HUDEP2 cells.

The above data do not address whether *Asf1a* performs a compensatory role upon *Asf1b* loss in H3.3 regulation and erythropoiesis. To investigate this question, we used CRISRP–Cas9 to delete *Asf1a, Asf1b*, and *Hira* with two sgRNAs for each gene in Murine erythroleukemia (MEL) cells (Fig. 3A and B, and Supplementary Figs S6–S8). As expected, loss of *Asf1a, Asf1b*, and *Hira* resulted in decreased protein level of H3.3 in uninduced and induced cells (Fig. 3C). H3K27ac, a hallmark of active enhancers and promoters, was reduced upon loss of *Asf1a, Asf1b*, and *Hira* as observed in H3.3 deletion [21, 40, 41], while other active histone marks H2A.Z and H3K9ac showed no significant changes (Fig. 3C).

We performed RNA-seq to further clarify whether *Asf1a, Asf1b*, and *Hira* play integrated or distinct roles in H3.3-encoding genes and erythroid genes (Fig. 3D). Compared to *Hira*, loss of *Asf1b* or *Asf1a* caused more similar transcriptional changes especially in induced cells, suggesting potential functional compensation between ASF1 family members (Supplementary Fig. S9A–C). Down-regulated genes upon deletion of *Asf1a, Asf1b*, and *Hira* were enriched in pathways related to erythroid differentiation, histone modification, and chromatin remodeling (Fig. 3E, and Supplementary Fig. S9D and E). Interestingly, there was more overlap between genes regulated by *Asf1a* and *Asf1b* than any other two factors among the top 10 DEGs associated with erythroid differentiation, histone modification, and chromatin remodeling pathways (Fig. 3E, heatmaps). Notably, erythroid genes *Hba-a, Id2, Gata2, Sox6*, and *Slc4a1*, as well as *H3f3a* were downregulated more by KO of *Asf1b* or *Asf1a* than by KO of *Hira* (Fig. 3E and Supplementary Fig. S9F). In contrast, *H3f3b* was upregulated in a potentially compensatory response to the loss of all three genes (Supplementary Fig. S9F).

We further generated the *Asf1a* and *Asf1b* double KO MEL cells (Supplementary Fig. S10A–D). KO of *Asf1a* or *Asf1b* individually decreased their gene expression but elevated expression of the remaining isoform, likely a compensatory response, seen also between *H3f3a* and *H3f3b* in *Asf1a/Asf1b* double KO cells (Fig. 3F and G). Double KO cells expressed moderately but significantly (*P *< 0.05) lower expression levels of erythroid and epigenetic genes than were downregulated by loss of *Asf1a* or *Asf1b* individually, except that DKO2 did not differ significantly from the single KOs for *Mllt3* or *Rbbp5* gene (Supplementary Fig. S10E). By contrast, Hira gene expression was significant upregulated in two DKOs, suggested that limited reduction of gene expression for the DKOs may result from the compensation by HIRA. The global protein levels of H3.3 and H3K27ac were strongle reduced in the double KO compared to any single KO (Supplementary Fig. S10F). To ask whether H3.3 is a main target of Asf1 proteins, we established H3f3a and H3f3b double KO MEL cells and found that expression of many erythroid genes was impaired upon loss of H3.3, similar to the changes in *Asf1b* and *Asf1b* double KO cells (Fig. 3H and Supplementary Fig. S11). These data support that *Asf1b* and *Asf1a* play compensatory roles in H3.3 and erythroid gene regulation.

To confirm the conservation of functional similarity between mouse and human ASF1 proteins, we conducted shRNA-mediated knockdown (KD) of *ASF1A* and *ASF1B* in HUDEP2 cells (Fig. 3I and Supplementary Fig. S12A). Similar to MEL cells, depletion of ASF1A and ASF1B showed functional similarities in regulation of epigenetic modification and erythroid homeostasis (Fig. 3I and L, and Supplementary Fig. S12B–D). The reciprocal compensatory effect of *ASF1A*/*ASF1B* and *H3F3A*/*H3F3B* gene expression after depletion of any one ASF1 family member confirms at least partial redundancy of ASF1A with ASF1B in regulation of H3.3 and erythroid gene expression.

### ASF1B modulates H3.3 nucleosomes globally at erythroid promoters and enhancers

**Figure 4.**
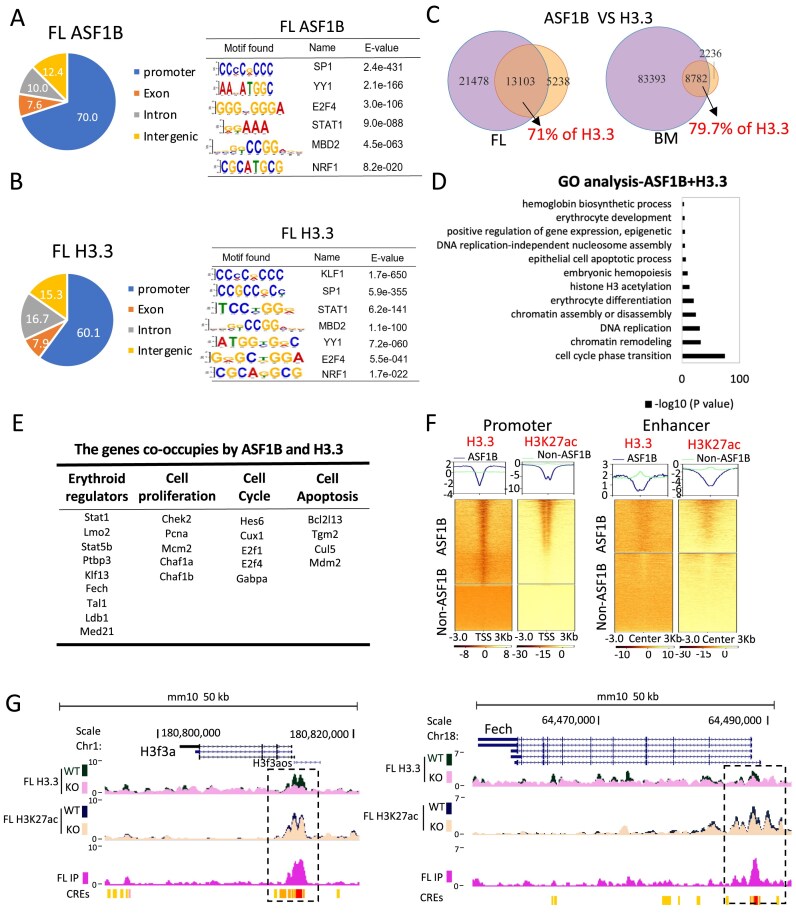
ASF1B is important for accumulation of histone H3.3 and H3K27ac at gene promoters and enhancers. (**A** and **B**) Left, ChIP-seq with HA and H3.3 antibody showing the percentage of ASF1B peaks (fetal liver, FL ASF1B) and H3.3 enrichment (FL H3.3) in E14.5 liver cells at different genomic features. Right, Top motifs from *Meme* search of ASF1B and H3.3 enrichment sites from ChIP-seq data. (**C**) Venn diagram of global overlapped sites between ASF1B and H3.3 in E14.5 liver cells (FL ASF1B versus H3.3) and bone marrow cells (BM ASF1B versus H3.3). (**D**). GO terms analysis of co-occupied peaks between ASF1B and H3.3. (**E**). The promoters of genes functioning as regulators of erythroid cell, cell proliferation, cell cycle, and cell apoptosis are enriched with ASF1B and H3.3 peaks. (**F**) Heatmap displaying differential signals of H3.3 and H3K27ac ChIP-seq when *Asf1b* KO compared to WT E14.5 liver cells in two categories (ASF1B-binding or non-ASF1B binding) at promoter (left) and enhancer (right). Left, each row represents a 3 kb window centered at Transcription Start Site (TSS). Right, each row represents a 3 kb window centered around the enhancer. (**G**) Screen shot showing ASF1B ChIP-seq, changed H3.3 and H3K27ac ChIP-seq signals at *H3f3a* and *Fech* gene loci in E14.5 liver cells. CREs indicated *cis*-regulatory elements as promoter (red) and enhancer (yellow).

To investigate a direct role of ASF1B in H3.3 chromatin, we performed ASF1B and H3.3 ChIP-seq in C-terminal HA-tagged ASF1B mice generated by CRISPR-CAS9 (Supplementary Fig. S13). About 70% of the HA (ASF1B) peaks in E14.5 liver cells were located at promoters, while 22.4% were found in intergenic or intronic regions, suggesting that ASF1B might function at potential enhancer sites (Fig. 4A). Notably, ASF1B peaks were significantly enriched for motifs associated with erythroid transcription factors SP1/KLF1, E2F4, STAT1, and epigenetic factor MBD1 (Fig. 4A). ASF1B maintained stable, similar genome-wide occupancy through to the adult bone marrow cells (Supplementary Fig. S14A–C).

In general, H3.3 peaks exhibited a similar genomic distribution (60% at promoters and 32% at potential enhancers) and motif enrichment compared to ASF1B in E14.5 fetal liver cells (Fig. 4B). At the embryonic stage, 71% (13 103) of H3.3 peaks were co-occupied by ASF1B (Fig. 4C). In contrast, more than half of the H3.3 peaks (55.9%) switched to locating at potential enhancers at the adult stage (Supplementary Fig. S14A). However, 79.7% of H3.3 peaks were still occupied by ASF1B (Fig. 4C), supporting the functional collaboration of ASF1B and H3.3 through development.

ASF1B and H3.3 overlapped peaks were enriched at promoters of genes involved in pathways of epigenetic regulation, hemoglobin biosynthesis, erythrocyte development, DNA replication, and cell cycle (Fig. 4D and E). This supports a direct partnership between ASF1B and H3.3 in erythroid and epigenetic gene expression. Accordingly, loss of *Asf1b* resulted in significant reduction of H3.3 enrichment at promoters, such as ASF1B-regulated genes *H3f3a, Fech*, and *Lmo2*, which was accompanied by decreased H3K27ac in fetal liver cells (Fig. 4F and G, and Supplementary Fig. S14D and E). At enhancers, the reduction of H3.3 enrichment was modest at the fetal stage but became remarkable genome wide at the adult stage (Fig. 4F and Supplementary Fig. S14F). For examples, distal enhancers of the *Lmo2* gene exhibited decreased H3.3 level upon loss of Asf1b across the fetal to adult development (Supplementary Fig. S14D). In contrast, H3K27ac did not show significant reduction at the adult stage (Supplementary Fig. S14G–I). Thus, ASF1B is important for erythropoiesis globally by exerting a profound effect on H3.3 enrichment for the activity of promoters and enhancers.

### ASF1B regulates H3.3 enrichment and chromatin accessibility to repress embryonic/fetal globin gene expression

**Figure 5.**
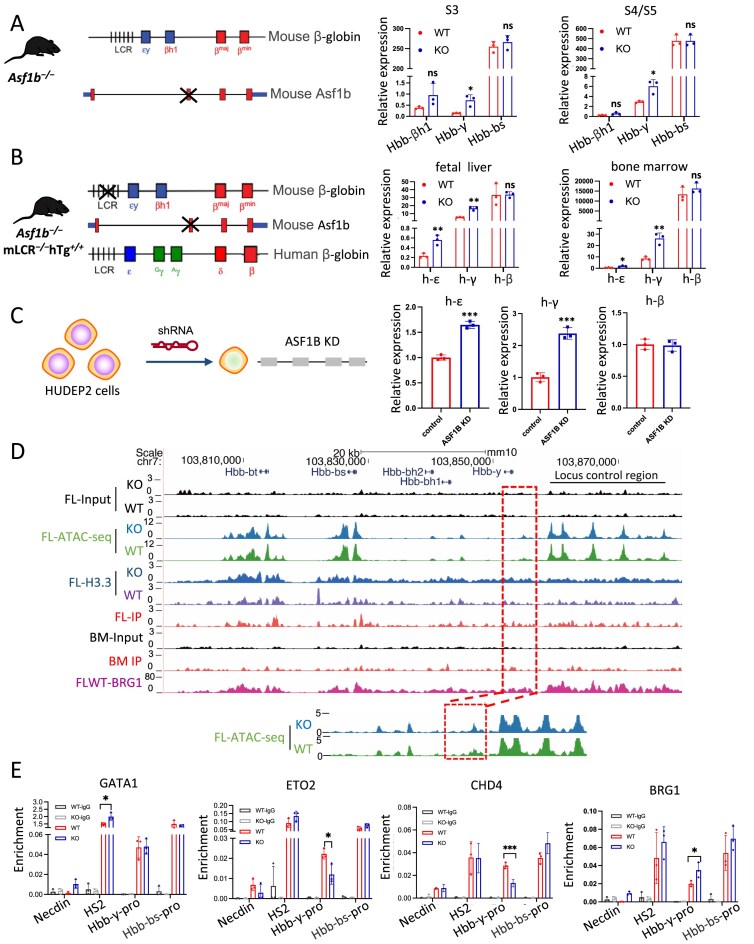
ASF1B regulates embryonic/fetal globin gene expression by influencing the H3.3 enrichment and the occupancy of chromatin remodelers. (**A**) Schematic diagram of β-globin locus in *Asf1b*^−/−^ mouse (left panel). RT-PCR showing globin gene expression in S3 and S4/S5 populations in *Asf1b ^−/−^* and WT E14.5 liver cells (right panel). (**B**) Schematic diagram of mouse and human β-globin locus in *Asf1b^−/−^* mLCR*^−/−^* hTg*^+/+^* mouse that carried a human β-globin transgene (left panel). RT-PCR showing human epsilon (ε), gamma (γ), and beta (β) globin gene expression in E14.5 liver and bone marrow cells of KO (*Asf1b^−/−^* mLCR*^−/−^* hTg*^+/+^*) mice and WT (mLCR*^−/−^* hTg*^+/+^*) littermates (right panel). (**C**) Schematic diagram of shRNA-mediated KD of *ASF1B* gene in HUDEP2 cells (left panel). RT-PCR showing globin gene expression in *ASF1B* KD and control HUDEP2 cells (right panel). (**D**) Screen shots of β-globin locus showing ChIP-seq signals of ASF1B in fetal liver (FL IP) and in bone marrow (BM IP) cells, as well as H3.3 and ATAC-seq signal in *Asf1b ^−/−^* and WT mice. (**E**) ChIP-qPCR for GATA1, ETO2, CHD4, and BRG1 occupancy in the β-globin locus in *Asf1b ^−/−^* and WT E14.5 liver cells. Error bars in panels (A), (B), (C), and (E) represent SD. *N* = 3 biological replicates. *P < 0.05, **P < 0.01, ***P < 0.001 by two-tailed Student’s *t-*test.

Previous studies indicated that persistence of embryonic *Hbb-y* globin gene expression in adult mouse erythroid tissues where it is normally silenced was associated with deletion of ASF1B [42]. However, the mechanism underlying this observation remained unclear. We established different models to explore the potential repression of globin genes by ASF1B (Fig. 5A–C). RNA-seq and RT-qPCR revealed sustained elevation of embryonic *Hbb-bh1* and *Hbb-y* globin gene expression upon loss of *Asf1b* in E14.5 S0–S5 mouse fetal liver cells (Fig. 5A and Supplementary Fig. S15A). We crossed *Asf1b^−/-^* animals with mice homozygous for a single copy of a human β-globin locus transgene and deletion of the endogenous mouse LCR, creating an *in vivo* model to investigate human globin genes (Fig. 5B, left). Upregulation of human embryonic (ε-) and fetal (γ-) globin genes was observed in the *Asf1b^−/−^* mLCR*^−/−^* hTg*^+/+^* fetal liver and bone marrow cells in comparison to controls (Fig. 5B, right). Accordingly, in HUDEP2 cells and K562 cells, KD or KO of ASF1B resulted in the increased expression of ε- and γ- globin. The results strongly support a repressive role of ASF1B in regulation of human embryonic/fetal globin genes (Fig. 5C and Supplementary Fig. S15B–E).

ChIP-seq showed Asf1b loss not only enhanced H3.3 enrichment but also strengthened ATAC-seq signals upstream of the embryonic *Hbb-y* globin gene promoter in fetal liver cells (Fig. 5D). In addition, loss of ASF1B increased the ASF1A occupancy in *Hbb-y* globin gene promoter, revealing at least a partial compensatory role upon ASF1B loss (Supplementary Fig. S15F and G). These results implicated a requirement for ASF1B in the chromatin state of the gene promoter.

To investigate whether erythroid transcription factors were involved in epigenetic regulation of ASF1B, we performed ChIP-qPCR to check occupancy of the GATA1 complex at the globin locus in the presence or absence of ASF1B. GATA1 exhibited increased enrichment at the LCR, accompanied by a decrease of the co-repressor ETO2 at the *Hbb-y* promoter upon deletion of ASF1B (Fig. 5E). However, the looping mediator LDB1 was unchanged at the β-globin locus (Supplementary Fig. S15H). We previously reported antagonism between activator Hemogen/BRG1 complexes and repressive ETO2/NuRD complexes in regulation of globin gene expression [43]. Here, we detected down-regulation of CHD4 (NuRD complex ATPase) and up-regulation of BRG1 occupancy at the *Hbb-y* promoter in *Asf1b^−/−^* cells compared to controls (Fig. 5E). Together, the results suggest that ASF1B modulates the epigenetic environment of globin genes through regulation of H3.3 and key transcription factor occupancy involved in chromatin opening.

### ASF1B interacts with the histone modifier BRG1 for H3.3 deposition and active chromatin establishment

**Figure 6.**
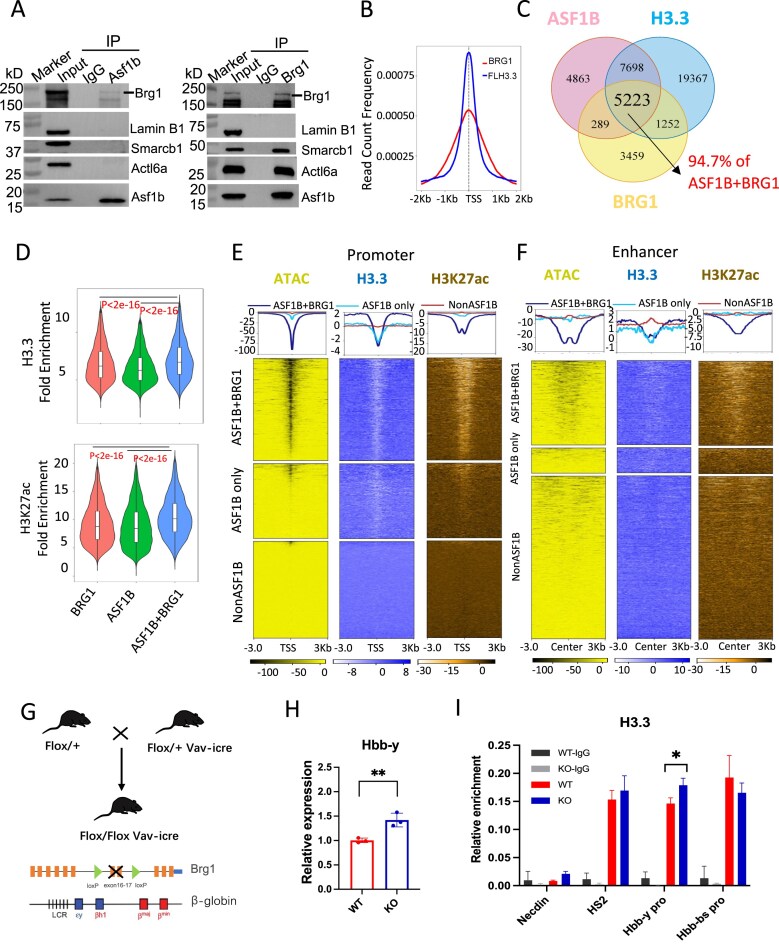
ASF1B recruits BRG1 to maintain H3.3 enrichment and chromatin accessibility for gene expression. (**A**) Co-IP analysis of interactions between ASF1B and SWI/SNF complex components Brg1 (the band on the top), Smarcb1 and Actl6a in E14.5 liver cells. (**B**) Peak density map of H3.3 and BRG1 peaks aligned at TSS at promoters with ASF1B (FL HA) peaks in E14.5 liver cells. (**C**) Venn diagram of global overlapped binding sites among ASF1B, BRG1, and H3.3 in E14.5 liver cells. (**D**) Violin Plot showing the enrichment of H3.3 and H3K27ac at binding sites of BRG1, ASF1B, and overlapped sites between them (BRG1 + ASF1B peaks, *P *< 2e-16). (**E** and **F**) Heatmap displaying changed H3.3 and H3K27ac ChIP-seq, as well as ATAC-seq signals between *Asf1b* KO and WT liver cells in three categories (BRG1 + ASF1B sites, ASF1B only or non-ASF1B binding sites) at promoter (left) and enhancers (right, determined by EnhancerAtlas for fetal liver cells). Left, each row represents a 3-kb window centered at TSS. Right, each row represents a 3-kb window centered around the enhancer. (**G**) Schematic diagram showing generation of *Brg1*^−/−^ mouse. (**H**) RT-PCR showing *Hbb-y* globin gene expression in of *Brg1 ^−/−^* and WT E14.5 liver cells. (**I**) ChIP-qPCR for H3.3 enrichment at the β-globin locus in *Brg1 ^−/−^* and WT E14.5 liver cells. In panels (H) and (I), error bars represent SD. *N* = 3 biological replicates. *P < 0.05, **P < 0.01 by two-tailed Student’s *t*-test.

To determine the mechanism by which ASF1B regulates the epigenetic environment, we conducted co-immunoprecipitation experiments using E14.5 liver cells. We detected a reciprocal interaction between ASF1B and SWI/SNF complex component BRG1. Notably, ASF1B interacted with BRG1, but not other SWI/SNF complex proteins Smarcb1 and Actl6a (Fig. 6A, the top band indicated BRG1). Previous studies indicated that BRG1 was recruited to erythroid gene promoters and enhancers to mediate DNA looping for gene activiation during differentiation [43–46]. We further found in our previous ChIP-seq studies [43] that BRG1-occupied sites were highly enriched for the motifs of erythroid transcription factors KLF1, STAT1, GATA5, YY1, and E2F4, overlapping with ASF1B binding motifs in E14.5 liver cells (Fig. 4A and Supplementary Fig. S16A). Overall, BRG1 shared >50% of peaks with ASF1B (ASF1B + BRG1 peaks), which were predominantly located at promoters of genes associated with erythropoiesis, cell proliferation, and cell cycle (Fig. 6B, and Supplementary Fig. S16B and C). Notably, 94% (5223 of 5512) of the ASF1B + BRG1 peaks were co-marked by H3.3 (ASF1B + BRG1 + H3.3) (Fig. 6C and D). These peaks were significantly enriched at genes in pathways related to erythrocyte differentiation and chromatin assembly or disassembly (Supplementary Fig. S16D). In addition to H3.3, ASF1B + BRG1 peaks were globally associated with enriched H3K27ac, and ATAC-seq signals compared to ASF1B or BRG1-only peaks at gene promoters and enhancers (Fig. 6D–F). These data suggest that ASF1B and BRG1 closely collaborate to regulate erythropoiesis by modulating H3.3, H3K27ac, and chromatin accessibility.

To test this idea, we set out to generate *Brg1* conditional KO mice. Genetic deletion of *Brg1* is lethal during the mouse pre-implantation stage [47]. In contrast, mice carring a Brg1 ENU-induced hypomorphic mutation survive embryogenesis but exhibit a postnatal developmental phenotype associated with runting and incompletely penetrant lethality [48, 49]. Conditional *Brg1* deletion had been conducted using Tie2-Cre to excise the gene both in endothelial cells (vasculature) and hematopoietic cells [50]. Mid-gestational lethality was observed due to failure to transcribe embryonic α- and β-globins and resultant anemia. This result suggests that BRG1 is an activator of embryonic globin transcription. We generated *Brg1* cKO mice by crossing Flox/+ mice with Flox/+ Vav-iCre mice (Fig. 6G). Vav-iCre gene excision is highly specific to the hematopoietic system, avoiding vasculature effects.

Although *Brg1* cKO mice exhibited significant anemia and fetal liver hypoplasia at E14.5, conditional deletion of *Brg1* resulted in the up-regulation of *Hbb-y* gene, and down-regulation of the *Lmo2* and *Fech* genes (Fig. 6H and Supplementary Fig. S17A–D). Thus, Hbb-y shows a similar upregulation upon either *Brg1* or *Asf1b* KO that was associated with increased H3.3 enrichment (Fig. 6I). In addition, *Lmo2* and *Fech* down-regulation was accompanied by reduced H3.3 occupancy at promoters in either *Brg1* or *Asf1b* KO cells (Supplementary Fig. S17D and E). In contrast, *Stat1* showed opposite changes in gene expression between the two KO cells, without significant H3.3 alteration (Supplementary Fig. S17E and F). The results support the idea that the effect of BRG1 and ASF1B on erythropoiesis may involve their co-regulation of H3.3 enrichment at erythroid gene promoters.

## Discussion

ASF1B plays a critical role in pancreatic β-cells proliferation, early embryogenesis, and tumorigenesis [8–15, 51, 52], but its function in erythropoiesis remains unclear. Erythropoiesis is a complex process that required close coordination between transcription factors, prominently GATA1, KLF1, NFE2, and RUNX1, and epigenetic regulators, establishing a cell-type specific transcriptome and chromatin landscape across development and differentiation [53–56]. For example, GATA1 recruits chromatin modifiers CBP/P300/BRG1 to mediate enhancer contact with target promoters for gene activation [46, 57]. By contrast, ETO2 is a key repressor for GATA1 negative function in gene expression through recruitment of the nucleosome remodeling and histone deacetylase (NuRD) complex [33, 58]. Here, we identified a unique role of ASF1B in modulating H3.3 gene expression and chromatin occupancy of this histone variant, specifically in embryonic erythropoiesis. ASF1B emerges as a pivotal factor with the cooperation of BRG1 for active chromatin, involved in the control of H3.3 enrichment, H3K27ac enrichment and chromatin accessibility in the erythroid genome.

HIRA is well-known for depositing H3.3 in transcriptionally active genes [51, 59, 60]. Surprisingly, *Hira*-KO mice exhibit normal fetal hematopoiesis and erythropoiesis [23, 24]. Unlike *Hira, Asf1b* prominently influenced the expression of H3.3-encoding genes *H3f3a*/*H3f3b* as well as the key regulators of H3.3 chromatin assembly during mouse erythroid development (Figs 2E, H, I and 3G). The role of ASF1B in H3.3 enrichment at erythroid genes across development differs between embryonic and adult erythropoiesis, as there appears to be a shift in H3.3 deposition from gene promoters (decreased by 23.7%) to enhancers (increased by 24%) at the different stages (Fig. 4A–C and Supplementary Fig. S14A and B). Loss of ASF1B had a more profound impact on H3.3 enrichment at the promoter region during the embryonic stage but had an equal effect on both promoters and enhancers during the adult stage (Fig. 4F and G, and Supplementary Fig. S14D–G). These data strongly support the regulatory role of ASF1B in H3.3 deposition during development. However, 70% of ASF1B binding sites were maintained at promoters throughout the fetal and adult stages (Fig. 4A–C, and Supplementary Fig. S14A and B). Beyond ASF1B binding sites, non-ASF1B binding sites were also associated with reduced H3.3 enrichment upon loss of *Asf1b* in adult bone marrow cells (Supplementary Fig. S14D), indicating the direct and indirect regulation of H3.3 by ASF1B. However, the complexity of ASF1B function in adult hematopoiesis, whether HIRA-dependent or independent, requires further investigation.

Our results suggest that it is highly likely that the effect of ASF1B on H3.3 enrichment and erythropoiesis have similarities to ASF1A. Our RNA-seq data in mice revealed increased *Asf1a* expression following a reduction of *Asf1b* expression (Fig. 2E and Supplementary Fig. S4J). Consistent with this, RNA-seq data from MEL and HUDEP2 cells revealed a high degree of similarity in the erythroid transcriptomes regulated by ASF1A and ASF1B (Supplementary Figs S9B and C, and 3J). Generally, ASF1A and ASF1B play distinct functional roles in chromatin assembly [5, 9, 16]. However, our study revealed that ASF1A maintains moderate expression levels across different stages of development and various types of erythroid cells. This may act as a compensatory force, effectively counteracting the impact of ASF1B deficiency on H3.3 gene/protein level and erythropoiesis (Fig. 3F and G, and Supplementary Fig. S10E and F). H3F3A and H3F3B also exhibit compensatory effects in response to ASF1B-related genetic deficiencies. Therefore, the functional redundancy between ASF1A/ASF1B and H3F3A/H3F3B may be responsible for the mild phenotypic changes observed in ASF1B-deficient mice [42]. Furthermore, increased ASF1A occupancy after loss of ASF1B might ensure H3.3 enrichment at gene promoters to shape the erythroid transcriptome during development.

To investigate the mechanisms underlying the effect of ASF1B on embryonic and fetal globin genes, we established *in vitro* and *in vivo* models (Fig. 5A–C). Previous studies have showed that the histone variants H3.3 and H2A.Z are incorporated into the β-globin locus during transcription activation via different mechanisms [21]. Consistently, we found that ASF1B repression of globin gene expression was mediated by epigenetic and erythroid transcription factors independent of H2A.Z (Fig. 3C). De-repression of globin genes upon ASF1B deletion was primary associated with epigenetic environmental changes such as increased H3.3 enrichment and greater chromatin accessibility at promoters (Fig. 5D). GATA1/BRG1-ETO2/CHD4 antagonism appears to play an essential role in the ASF1B regulatory network governing globin gene expression (Fig. 5E), which is independent of another key repressor, KLF1, as previous reported [61]. Previous studies have shown that H3.3 recruits chromatin remodeling complexes, particularly SWI/SNF and NuRD, which alter nucleosome dynamics at regulatory elements and affect transcription factor binding [41, 62, 63]. However, in erythroid cells, we found that ASF1B/H3.3 preferentially recruited BRG1 to erythroid gene promoters as opposed to enhancers (Fig. 6E). Together, these studies support our hypothesis that the influence of ASF1B/H3.3 on chromatin states is a key mechanism underlying ASF1B’s roles in erythropoiesis and gene expression.

Our examination of the functional cooperation between ASF1B and BRG1 revealed that H3.3 enrichment, rather than H3K27ac enrichment or ATAC peaks, was specifically targeted to overlapped sites of ASF1B and BRG1 at gene promoters (Fig. 6C, E, and F). Although BRG1 contributes to chromatin accessibility, its regulation of erythroid gene expression was more strongly associated with H3.3 enrichment. This suggests that H3.3 is one of the most important elements in ASF1B/BRG1 function. The role of H3.3 in maintaining active transcription remains controversial [40, 41, 64–66]. However, our data support the hypothesis that H3.3 facilitates chromatin accessibility and maintains epigenetic memory in an activated state. Therefore, H3.3 enrichment mediated by ASF1B is a hallmark of an active chromatin environment, which is necessary for proper transcriptomic regulation during erythropoiesis.

**Figure 7.**
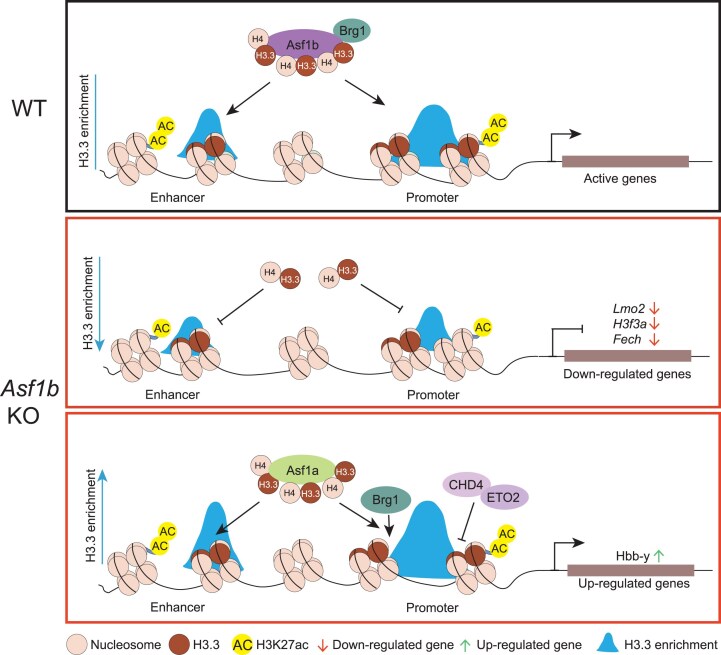
Model depicting ASF1B-H3.3 function in gene expression during erythropoiesis. In normal mouse fetal liver, Asf1b recruits Brg1 to orchestrate H3.3 and H3K27ac enrichment at promoters to regulate the expression of erythroid and epigenetic genes. KO of Asf1b resulted in the distinct changes of H3.3 enrichment on these genes. *Lmo2, H3f3a*, and *Fech* genes were downregulated upon KO of Asf1b, accompanied by decreased enrichment of H3.3 and H3K27ac. In contrast, the Hbb-y gene was up-regulated upon ASF1B loss, with increased H3.3 and chromatin accessibility at the promoter likely due to the compensatory increase of ASF1A occupancy. The deceased occupancy of ETO2/CHD4 and increased BRG1 likely contribute to the increased Hbb-y gene expression after loss of Asf1b.

In summary, our study reveals a unique regulatory pathway for H3.3 enrichment, which is mediated by ASF1B during erythropoiesis. It particularly highlights the cooperative role of ASF1B and BRG1 in H3.3 enrichment and chromatin accessibility during the embryonic stage (Fig. 7). These findings provide new insights into the mechanisms by which ASF1B maintains active chromatin dynamics to promote erythroid gene expression. Notably, ASF1B repression of embryonic/fetal globin gene expression could be considered a new target for treating β-globin hemoglobinopathies.

## Supplementary Material

gkag447_Supplemental_File

## Data Availability

RNA-seq, ChIP-seq, and ATAC-seq data are available at GEO (https://www.ncbi.nlm.nih.gov/geo/) under accession numbers GSE215076, GSE214528, and GSE285838.
