# Supplementary material for: ASF1B promotes erythropoiesis by regulating the establishment and enrichment of H3.3 nucleosomes
Source: Nucleic Acids Res. 2026 May 8;54(9):gkag447. doi: 10.1093/nar/gkag447 (PMC13153709; doi:10.1093/nar/gkag447)
Supplement: gkag447_Supplemental_File [file gkag447_supplemental_file.pdf]

SUPPLEMENTARY MATERIALS

1. Supplementary Materials and Methods

2. Supplementary Tables

3. Supplementary figures and Legends

SUPPLEMENTARY MATERIALS AND METHODS

Generation of Asf1b HA-tag Mice by CRISPR-Cas9

Mouse protocols were approved by the NIDDK Animal Care and Use Committee in accordance with AALAC specifications. crRNA and tracrRNA (IDT) were separately dissolved in nuclease-free duplex buffer (IDT) at a concentration of 200 μM. The RNAs were mixed in equal molar quantity, heated at 95 °C for 5 minutes, and cooled down to the room temperature, forming a duplex guide RNA (gRNA). The gRNA, Cas9 nuclease (IDT), and a single strand DNA (ssDNA) were added to Opti-MEM medium (Thermo Fisher) to make a working solution (8 μM gRNA, 300 ng/μl Cas9 nuclease, 2 μg/μl ssDNA) for mouse zygote electroporation. Zygotes were collected from superovulated B6D2F1 mice and incubated in EmbryMax KSOM medium (Millipore Sigma) at 37 °C, 5% CO_2. After several washes in Opti-MEM, the embryos were transferred to the prepared gRNA/Cas9/ssDNA woking solution (in a total volume of 50μl) in a chamber of a CUY505P electrode (Bulldog Bio), and electroporated by using a NEPA21 Electro-Kinetic Transfection System (Bulldog Bio). The electroporated embryos were washed in EmbryMax KSOM medium and incubated in the EmbryMax KSOM medium overnight at 37 °C, 5% CO_2. To obtain live Asf1b-HA mice, two-cell embryos formed after the overnight incubation were transferred to oviduct of pseudo-pregnant ICR mice. The sequence of crRNA, ssDNA, and primers for genotyping the KI mice are listed in Supplementary Table 1.

Data Analysis

RNA-seq

<u>E14.5 mouse fetal liver cells and bone marrow cells</u>

In E14.5 fetal liver cells, a total of 30 stranded libraries were sequenced. 3 replicates were conducted for RNA-seq of each treatment, include S0, S1, S2, S3 and S4/5 population in both WT and *Asf1b* KO cells.The sequencing was performed by using 51-bp single-end reads on an Illumina HiSeq 2500. The treatment between 56.3 and 89.7 million reads were produced, depending on the replicate.

In bone marrow cells, a total of 24 stranded libraries were sequenced. 3 replicates were conducted for RNA-seq of each treatment, include E1, E2, E3 and E4 population in both WT and *Asf1b* KO

cells.The sequencing was performed by using 51-bp single-end reads on an Illumina HiSeq 2500. The treatment between 7.6 and 108.1 million reads were produced, depending on the replicate.

Asf1a, *Asf1b* and *Hira* KO MEL cells

In MEL cells, a total of 16 stranded libraries were sequenced in *Asf1a*, *Asf1b* and *Hira* KO experiments. 2 replicates were conducted for each treatment, include control, *Asf1a* KO, *Asf1b* KO, *Hira* KO at uninduced and DMSO induced situation, respectively. The sequencing was performed by using paired-end reads on an Illumina Novaseq X Plus. Between 19.9 and 28.6 million reads were produced, depending on the replicate.

ASF1B and *ASF1A* KD HUDEP2 cells

In HUDEP2 cells, a total of 6 stranded libraries were sequenced in *ASF1B* and *ASF1A* KD experiments, which include 2 replicates for control, 2 for *ASF1B* KD and 2 for *ASF1A* KD by using paired-end reads on an Illumina Novaseq X Plus. Between 22.3 and 35.7 million reads were produced, depending on the replicate.

ASF1B KD $CD34^+$ cells

In $CD34^+$ cells, a total of 4 stranded libraries were sequenced in *ASF1B* KD experiments, which include 2 replicates for control and 2 for *ASF1B* KD by using paired-end reads on an Illumina Novaseq X Plus. Between 18.6 and 20.1 million reads were produced, depending on the replicate.

Raw sequence reads from RNA-seq in mouse fetal liver cells, bone marrow cells, MEL cells, human HUDEP2 cells were trimmed with fastp v0.23.4 (Chen et al., 2018) to remove any adapters. Sequencing library quality was assessed and the presence of common sequencing contaminants was evaluated with fastp v0.23.4 (Chen et al., 2018) with default parameters.

Trimmed reads were mapped to *Mus musculus* GENCODE release M25 and the *Homo sapiens* GENCODE release 47 assembly using hisat2 v2.2.1 (Kim et al., 2015) with default parameters. Multimapping reads were filtered using samtools v1.6 (Li et al., 2009). Uniquely aligned reads were then mapped to gene features using Subread featureCounts v2.0.6 (Liao et al., 2014) using stranded option S2 with default parameters. Differential expression between groups of samples

was tested using R version v4.2.1 (R Core Team, 2018) (R Core Team) with DESeq2 v1.38.3(Love et al., 2014).

GO analysis /KEGG pathway enrichment (Figure 2B, 2G, 2J, S4B, S4C, S5F, S5G 3E, 3K and S9E) was performed by clusterProfiler v4.6.2.

Volcano plot (Figure 2A, 2F, 2I , S4D, S4E, S4F, S4G, S4H, S5B, S5C, S5D, S5E and S9D) was produced by customized scripts with ggplot2 v3.5.0 packages based on the differential expression analysis of DESeq2 v1.38.3.

PCA analysis (Figure 3D, S4A, S5A and S12B) was produced by customized scripts with DESeq2 v1.38.3.

The lfc-lfc plot (Figure 2D, 3I, S9B and S9C) was generated by ggplot2 v3.5.0.

Venn diagram (Figure S4K, S4M, S5H, S5J and 3L), Heatmap (Figure 1A, 2C, S4J, 3E, S9A and S12D) and UpSet plot (Figure S4L, S4N, S4I and S4K) was generated by TBtools v2.142.

ChIP-seq in E14.5 fetal liver cells and bone marrow cells

ChIP-seq in WT E14.5 fetal liver cells, a total of 8 libraries, 2 inputs and 2 IP for each antibody (HA, H3.3 and H3K27ac), were sequenced and produced 39.2 to 62.7 million reads depending on replicate. For H3.3 and H3K27ac ChIP-seq in ASF1B KO E14.5 fetal liver cells, a total of 6 libraries, 2 inputs and 2 IP for each antibody, were sequenced and produced 40.1 to 70.3 million reads depending on replicate.

ChIP-seq in WT bone marrow cells, a total of 8 libraries, 2 inputs and 2 IP for each antibody (HA, H3.3 and H3K27ac), were sequenced and produced 39.5 to 51.7 million reads depending on replicate. For H3.3 and H3K27ac ChIP-seq in ASF1B KO bone marrow cells, a total of 6 libraries, 2 inputs and 2 IP for each antibody, were sequenced and produced 35.5 to 77.6 million reads

depending on replicate.

Reads were trimmed as described above and aligned to the mouse GENCODE release 18 but using Bowtie2 v2.3.5 (Langmead and Salzberg, 2012). Multimapping reads were removed with samtools v1.9 using the "view" subcommand and the additional argument "-q 20", and duplicate reads were removed with the Picard v2.21.4 (Toolkit, 2019) Mark Duplicates tool. BigWig signal tracks were created with deepTools v3.3.0 (Ramirez et al., 2016) using the bamCoverage tool. Peaks were called for each replicate individually using macs2 2.2.7.1 (Zhang et al., 2008). IDR analysis was performed by IDR Filter v1.2.

ASF1B (HA) and H3.3 distribution pie chart (Figure 4A, 4B and S14A) were produced by ChIPSeeker.

Venn diagram of overlapped peaks among ASF1B (HA), H3.3 and H3K27ac (Figure4C, S14B, 6C, S16B) was performed by ChIPpeakAnno v3.14.2

Motif analysis of ASF1B, H3.3 binding sites (Figure 4A, 4B, S14A and S16A) was performed by MEME-ChIP v5.1.0.
Aggregation plot for the ASF1B (HA) and H3.3 co-localization in fetal liver (FL) and bone marrow (BM) cells (Figure S14C), ASF1B (HA), H3.3 and BRG1 co-localization (Figure 6B) were generated by ChIPseeker v1.18.0.

Heatmap and profile plot of H3.3, H3K27ac differential binding in ASF1B KO / WT (Figure 4F, S114E) were generated by ChIPseeker v1.18.0 and deepTools 3.3.0
GO analysis /KEGG pathway enrichment (Figure 4D and Figure S16D) was performed by clusterProfiler v4.6.2.

ATAC seq data analysis
For ATAC-seq in WT and ASF1B KO E14.5 FL cell, a total of 4 libraries were sequenced and produced 49.0 to 63.1 million reads depending on replicate. The reads were trimmed as described above and aligned to the mouse GENCODE release 18 but using Bowtie2 v2.3.5 (Langmead and

Salzberg, 2012). Multimapping reads were removed with samtools v1.9 using the "view" subcommand and the additional argument "-q 20", and duplicate reads were removed with the Picard v2.21.4 (Toolkit, 2019) Mark Duplicates tool. BigWig signal tracks were created with deepTools v3.3.0 (Ramirez et al., 2016) using the bamCoverage tool.

The aligned reads were further converted to interval bed file format as an input for macs2 using SAMtoBED.py python script, which converts SAM alignment file to strand annotated bed files". Peaks were called for each replicate individually using macs2 2.2.7.1 (Zhang et al., 2008). Pooled peaks were separately called by providing both replicates simultaneously to macs2 with a command- "macs2 callpeak -t input_bed_file -f BEDPE -n NAME -g mm --keep-dup all --outdir out_put_directory --name sample_label". The bed files were converted to bigbed with ucsc-bedtobigbed for UCSC genome browser track.

Heatmap of ATAC-seq differential signals in WT/ASF1B KO (Figure 6E and 6F) were generated by deepTools 3.3.0

Integrated downstream analysis

To evaluate the effect of cooperativity among H3.3, H3K27ac and ATAC-seq signals on enhancer activity (Figure S14F, 6E and 6F), enhancers were defined in Enhancer Atlas (Gao and Qian, 2020). Genome browser views (Figure 4G, S14D, S14G, S14H and 5D) were produced by UCSC genome browser.

Violin Plot (Figure 6D) were produced using R package ggplot2.

References

Chen, S., Zhou, Y., Chen, Y., and Gu, J. (2018). fastp: an ultra-fast all-in-one FASTQ preprocessor. Bioinformatics *34*, i884-i890.

Gao, T., and Qian, J. (2020). EnhancerAtlas 2.0: an updated resource with enhancer annotation in 586 tissue/cell types across nine species. Nucleic Acids Res *48*, D58-D64.

Kim, D., Langmead, B., and Salzberg, S.L. (2015). HISAT: a fast spliced aligner with low memory requirements. Nat Methods *12*, 357-360.

Langmead, B., and Salzberg, S.L. (2012). Fast gapped-read alignment with Bowtie 2. Nat Methods *9*, 357-359.

Li, H., Handsaker, B., Wysoker, A., Fennell, T., Ruan, J., Homer, N., Marth, G., Abecasis, G., Durbin, R., and Genome Project Data Processing, S. (2009). The Sequence Alignment/Map format and SAMtools. Bioinformatics *25*, 2078-2079.

Liao, Y., Smyth, G.K., and Shi, W. (2014). featureCounts: an efficient general purpose program for assigning sequence reads to genomic features. Bioinformatics *30*, 923-930.

Love, M.I., Huber, W., and Anders, S. (2014). Moderated estimation of fold change and dispersion for RNA-seq data with DESeq2. Genome Biol *15*, 550.

R Core Team R: A language and environment for statistical computing. In R Foundation for Statistical Computing, Vienna, Austria. URL https://www.R-project.org/.

Ramirez, F., Ryan, D.P., Gruning, B., Bhardwaj, V., Kilpert, F., Richter, A.S., Heyne, S., Dundar, F., and Manke, T. (2016). deepTools2: a next generation web server for deep-sequencing data analysis. Nucleic Acids Res *44*, W160-165.

Toolkit, P. (2019). Broad institute, GitHub repository. See http://broadinstitute. github. io/picard.

Zhang, Y., Liu, T., Meyer, C.A., Eeckhoute, J., Johnson, D.S., Bernstein, B.E., Nusbaum, C., Myers, R.M., Brown, M., Li, W., et al. (2008). Model-based analysis of ChIP-Seq (MACS). Genome Biol *9*, R137.

SUPPLEMENTARY TABLES

Supplemental table 1-Antibodies

Name	Company	Catalog number
Anti-ASF1B	proteintech	22258-1-AP
Anti-ASF1A	proteintech	22259-1-AP
Anti-ASF1A	CellSignalingTechnology	2990T
Anti-H3F3B	Abclonal	A10220
Anti-HIRA	Abclonal	A1257
Anti-H3	Abclonal	A22348
Anti-H3.3	Abclonal	A4835
Anti-H3K27ac	Abclonal	A7253
Anti-H2AZ	Abclonal	A4599
Anti-H3K9ac	Abclonal	A7255
Anti-BRG1	Abclonal	A2117
Anti-SMARCB1	Abclonal	A3247
Anti-ACTL6A	Abclonal	A7709
Anti-LAMINB1	proteintech	12987-1-AP
Anti-HA	Abcam	ab9110
Anti-H3.3	Abcam	Ab176840
Anti-CBFA2T3(ETO2)	Abcam	ab33072
Anti-GATA1	Abcam	ab11852
Anti-LDB1	Abcam	ab96799
Anti-CHD4	Novus Biologicals	NB100-57519
Anti-BRG1	Abcam	ab110641
HRP Goat anti-Rabbit IgF (H+L)	Abclonal	AS014
VeriBlot	Abcam	ab131366
Ly-6G/Ly-6C Monoclonal Antibody (RB6-8C5) PE-Cyanine7	eBioscience	25-5931-82
CD11b Monoclonal Antibody (M1/70) PE-Cyanine7	eBioscience	25-0112-82
CD3e Monoclonal Antibody (145-2C11) PE-Cyanine7	eBioscience	25-0031-82
CD8a Monoclonal Antibody (53-6.7) PE-Cyanine7	eBioscience	25-0081-82
anti-mouse CD4 PE-Cyanine7	eBioscience	25-0041-82
CD45R (B220) Monoclonal Antibody (RA3-6B2) PE-Cyanine7	eBioscience	25-0452-82
CD19 Monoclonal Antibody (eBio1D3 (1D3)) PE-Cyanine7	eBioscience	25-0193-82
TER-119 Monoclonal Antibody	eBioscience	25-5921-82

(TER-119) PE-Cyanine7		
CD117 (c-Kit) Monoclonal Antibody (2B8) APC	eBioscience	17-1171-82
APC/Cy7 anti-mouse Ly-6A/E (Sca-1) Antibody	BioLegend	108126
PE anti-mouse CD150 (SLAM) Antibody	BioLegend	115904
FITC anti-mouse CD48 Antibody	BioLegend	103404
anti-mouse CD34 FITC	eBioscience	11-0341-82
PE Rat anti-mouse CD16/CD32 （FcrIII）	BD Biosciences	553145
Anti-mouse CD135 (Flt-3) PE	eBioscience	12-1351-82
APC Rat-Mouse TER-119	BD pharmingen	#557909
PE Rat Anti-Mouse CD71	BD pharmingen	#553267
Anti-Hu CD71	Invitrogen	2630409
Anti-Hu CD235a	Invitrogen	2565497

Supplemental table2-sgRNAs and primers sequences

Oligo name	Oligo sequence 5'-3'
mAsf1a sgRNA1	TTAGTGGGTCCCGTTCCTGC
mAsf1a sgRNA2	CTGATTACTTGCACCTACCG
mAsf1b sgRNA1	GCCCGGCCTCACCGTCAGAC
mAsf1b sgRNA2	CATAAATGATCTTCCACTCC
mHira sgRNA1	ACACCGCCATTGCTCCACAT
mHira sgRNA2	ATCACTTAGGTATTACAGAG
mH3f3a sgRNA	GATAGCGTCTGATTTCACGG
mH3f3b sgRNA	GAAGCCTCACCGCTACAGGT
hASF1A shRNA1	AGGCGTAACTGTTGTGCTAAT
hASF1A shRNA2	GTGAAGAATACGATCAAGTTT
hASF1B shRNA1	GAGTGGAAGATCATTTATGTT
hASF1B shRNA2	CTGGAGTGGAAGATCATTTATC
hH3F3B shRNA	GCTTCGAGAGATTCGTCGTTA
hH3F3A sgRNA	GTGCTTTGCAGGTAAAATGG
hASF1B sgRNA	AAGCTGATCTCGAACCGGAA
HA-KI mouse crRNA	CTCCATGGATTGCATCTGAC
HA-KI mouse ssDNA	AGACAGCTGGTGGTCCCCAAATCCTTGAGAT<br>GACCAAGCATGTCTGACTGAGGGCTTGTAG<br>GTCCCCTCCAGGTGTGCAAGGAGACTGCTGTG<br>GTCCTACTGAGCTCTACCTGGCTCCCCTGTC<br>AAGCGTAATCTGGAACATCGTATGGGTAGATG

	CAATCCATGGAGTTCTCAGGGAGGAGGCC TGGGATGCAACTAGG
mAsf1a F	GTGGTGCTGGATAACCCGTC
mAsf1a R	GGGACCCACTAAAACAGAGTCTA
mAsf1b F	TGGACAAGAGTTCATCCGTGT
mAsf1b R	CCAGTTGATATGGAATCGGGTC
mHira F	CCCACTGCTCAGATCATCGAA
mHira R	TGGGTTGAATTTCACAACAGTCA
mAsf1a sgRNA1 F	GGCTCTGCAGAAAGTGAAGAAT
mAsf1a sgRNA1 R	GGGTAGGGCTCTGAGGTAGTTT
mAsf1a sgRNA2 F	GCTATAGCTGCACTAACAGCCA
mAsf1a sgRNA2 R	TTTACTGGTGGGTTTTCCCTTA
mAsf1b sgRNA1 F	TCGCAAGTCACTGTGAAGAAGT
mAsf1b sgRNA1 R	GGAGGCGTATCTAATGGGAGAT
mAsf1b sgRNA2 F	TTGCCTAATGCTGACAAGAAGA
mAsf1b sgRNA2 R	GAAAGACGAACATATGCCTTCC
mHira sgRNA1 F	TCTGGTTTTGCCTTTTCTGTTT
mHira sgRNA1 R	GGCTACACAGAGAAACCCTGTC
mHira sgRNA2 F	AGGATGACGAGAAGGATGAAAA
mHira sgRNA2 R	GTCTTCTGCCGAATAACTCACC
mH3f3a F	TGTGGCCCTCCGTGAAATC
mH3f3a R	GGCATAATTGTTACACGTTTGGC
mH3f3b F	AAGCAGACCGCTAGGAAGTC
mH3f3b R	GTAACGACGGATCTCTCTCAGA
mLmo2 F	TCGGCCATCGAAAGGAAGAG
mLmo2 R	CGGTCCCCTATGTTCTGCTG
mGata2 F	CACCCCGCCGTATTGAATG
mGata2 R	CCTGCGAGTCGAGATGGTTG
mKdm5b F	AAGCCAAGCTCTGTTCAGCAA
mKdm5b R	GAAGGCAATCGTTCTTCTCACT
mMllt3 F	CCCGCCACCATTATTGAAAACT
mMllt3 R	TGCCTTGTCACATTCACCATTC
mRbbp5 F	TGGACAGAACTACCCAGAGGA
mRbbp5 R	AGTGTGCCCCACCTGTTAAAG
mH3f3a sgRNA F	GGAAACAACTGGCTACAAAAGC
mH3f3a sgRNA R	AACATCTTTTCCCCATAGCAAA
mH3f3b sgRNA F	CAGACCGCTAGGAAGTCCAC
mH3f3b sgRNA R	GGTAACGACGGATCTCTCTCAG
mSox6 F	AATGCACAACAAACCTCACTCT
mSox6 R	AGGTAGACGTATTTCGGAAGGA
mSlc4a1 F	AGATCCCAGATCGAGACAGC
mSlc4a1 R	GCTCCACATAGACCTGACCG
mAlas2 F	CTCCGAGGCATCTATGGCATC
mAlas2 R	ACACGAGGGTGTCTGCTTATG
mCited2 F	CCTCATGGGCGAGCACATAC
mCited2 R	GGTGCAAATCCGGCATGTAG

mId2 F	TCCGGTGAGGTCCGTTAGG
mId2 R	CAGACTCATCGGGTCGTCC
hASF1A-F	TGCTGGATAACCCTTCTCCTT
hASF1A-R	CAGGACCCACTAAAACAGAGTC
hASF1B-F	TCCGGTTCGAGATCAGCTTC
hASF1B-R	GTCGGCCTGAAAGACAAACA
hH3F3A sgRNA F	TTTTAAAGGCCTGGTACTGTGG
hH3F3A sgRNA R	ATAAGCACATGCAACAATTTGG
hH3F3A-F	TGTGGCGCTCCGTGAAATTAG
hH3F3A-R	CTGCAAAGCACCGATAGCTG
hH3F3B-F	GTGGCGCTTCGAGAGATTC
hH3F3B-R	GCGAGCCAACTGGATGTCTT
mEγ F	TGCTGACTGCTTTTGGAGAG
mEγ R	ACCAGCACATTACCCAAGAG
mβh1 F	AGAAGCTGGTGATTGGAGTG
mβh1 R	TCATAGACACATGGGATTGCC
mβmaj F	ACTCACAACCCCAGAAACAG
mβmaj R	GAGTTCACCTTTCCCCACAG
hγ globin F	TTCTGGAACGTCTGAGGTTATCAA
hγ globin R	GCCTTGTCCTCCTCTGTGAAAT
hβ globin F	AGCAACCTCAAACAGACACC
hβ globin R	AAAGAACCTCTGGGTCCAAG
hε globin F	CTACTCACTTTGGCAAGGAGTTCA
hε globin R	CGACAGCAGACACCAGCTTCT
mactin F	ACCCCATTGAACATGGCATT
mactin R	TGTAGAAGGTGTGGTGCCAGAT
hGADPPH F	ACCACAGTCCATGCCATCACT
hGADPPH R	CCATCACGCCACAGTTTCC
hASF1B sgRNA1 F	CAAGGTGTCGGTGCTGAA
hASF1B sgRNA1 R	GTTTCATGGGGGAGATCAAGT
HA-KI mouse F	CTCAGTTGCACTCCTGTTAAA
HA-KI mouse R	CCCAAATCCTTGAGATGACC
mNecdin F	AGCTCATGTGGTACGTGTTGGT
mNecdin R	GCTGCCCATGACCTCTTTCA
mHS2 F	CTGTGGACTTCCTCCTAGA
mHS2 R	TGAGGCTTAGGGTGTGT
mEγ-pro F	TGTTCTGACCCTTTGTTCTG
mEγ-pro R	AGAATACCTCCATATCTAATGTGC
m$\beta_{maj-pro}$ F	CAGGGAGAAATATGCTTGTCATCA
m$\beta_{maj-pro}$ R	GAGCAGATTGGCCCTTACCA

Supplementary figures and Legends

Figure S1

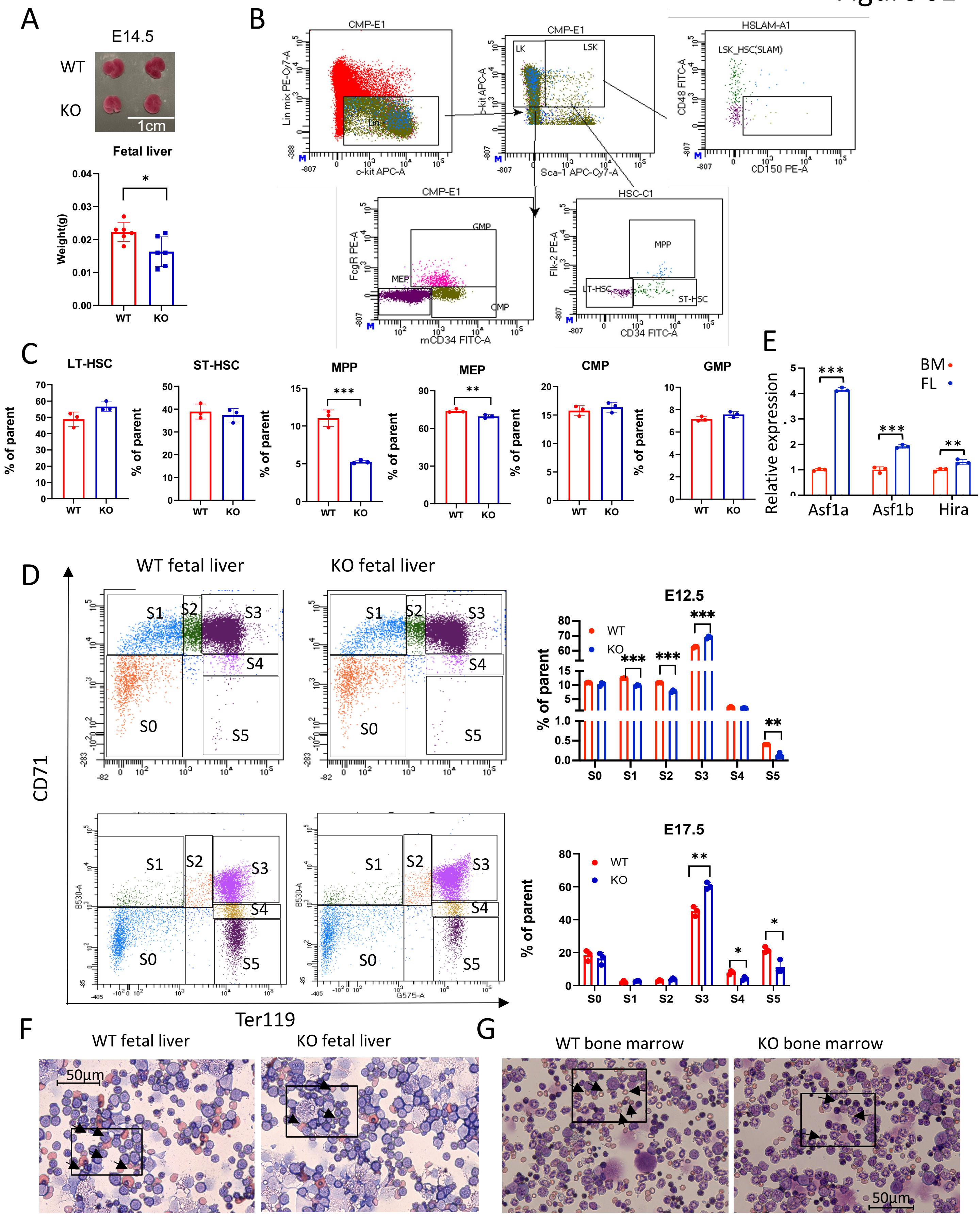

A
E14.5
WT
KO
1cm
Fetal liver
Weight(g)
WT
KO
B
CMP-E1
HSLAM-A1
HSC-C1
LK
LSK
LSK_HSC(SLAM)
GMP
MEP
CMP
MPP
LT-HSC
ST-HSC
Lin mix PE-Cy7-A
c-kit APC-A
Sca-1 APC-Cy7-A
CD48 FITC-A
CD150 PE-A
FcgR PE-A
mCD34 FITC-A
Flk-2 PE-A
CD34 FITC-A
C
LT-HSC
ST-HSC
MPP
MEP
CMP
GMP
% of parent
E
Relative expression
BM
FL
Asf1a
Asf1b
Hira
D
WT fetal liver
KO fetal liver
CD71
Ter119
S0
S1
S2
S3
S4
S5
E12.5
E17.5
% of parent
F
WT fetal liver
KO fetal liver
50μm
G
WT bone marrow
KO bone marrow
50μm

Figure S1. Loss of ASF1B impairs mouse erythropoiesis .

A. E14.5 fetal livers of *Asf1b*$^{-/-}$ mice have less cell numbers compared to control littermates. Scale bar, 1cm. **B**. Scheme of flow cytometry analysis of different populations of hematopoietic stem/progenitor cells (HSPCs) in WT and *Asf1b*$^{-/-}$ E14.5 liver cells. HSPC populations include LT-HSC (LSK CD34^{-} Flk2^{-}), ST-HSC (LSK CD34^{+}Flk2^{-}), MPP (LSK CD34^{+}Flk2^{+}), CMP (LK CD34^{+}FcγR^{-}), GMP (LK CD34^{+}FcγR^{+}), and MEP (LK CD34^{-} FcγR^{-}). **C.** Flow Cytometry Analysis showing the percentage of different populations of HSPCs (LT-HSC, ST-HSC, MPP, MEP, CMP, GMP) in WT and *Asf1b* $^{-/-}$ E14.5 liver cells. **D.** Flow cytometry with Ter119 and CD71 staining to separate maturational stages S0-S5 in E12.5 and E17.5 liver cells. **E**. Relative expression of *Asf1b*, *Asf1a* and *Hira* measured by RT-qPCR in E14.5 FL and bone marrow (BM) cells. **F-G.** Wright-Giemsa showing less mature red cells of *Asf1b* $^{-/-}$ E14.5 liver cells (F), *Asf1b* $^{-/-}$ BM cells (G) compared to controls. Bar, 50μm. Black arrows indicate the mature red cells. The cells in black rectangle are shown in Figure 1F-1G. Error bars in panels **A, C, D** and **E** represent SD. N=3 biological replicates. * *P*<0.05, ** *P*<0.01, *** *P*<0.001 by two-tailed Student's t test.

Figure S2

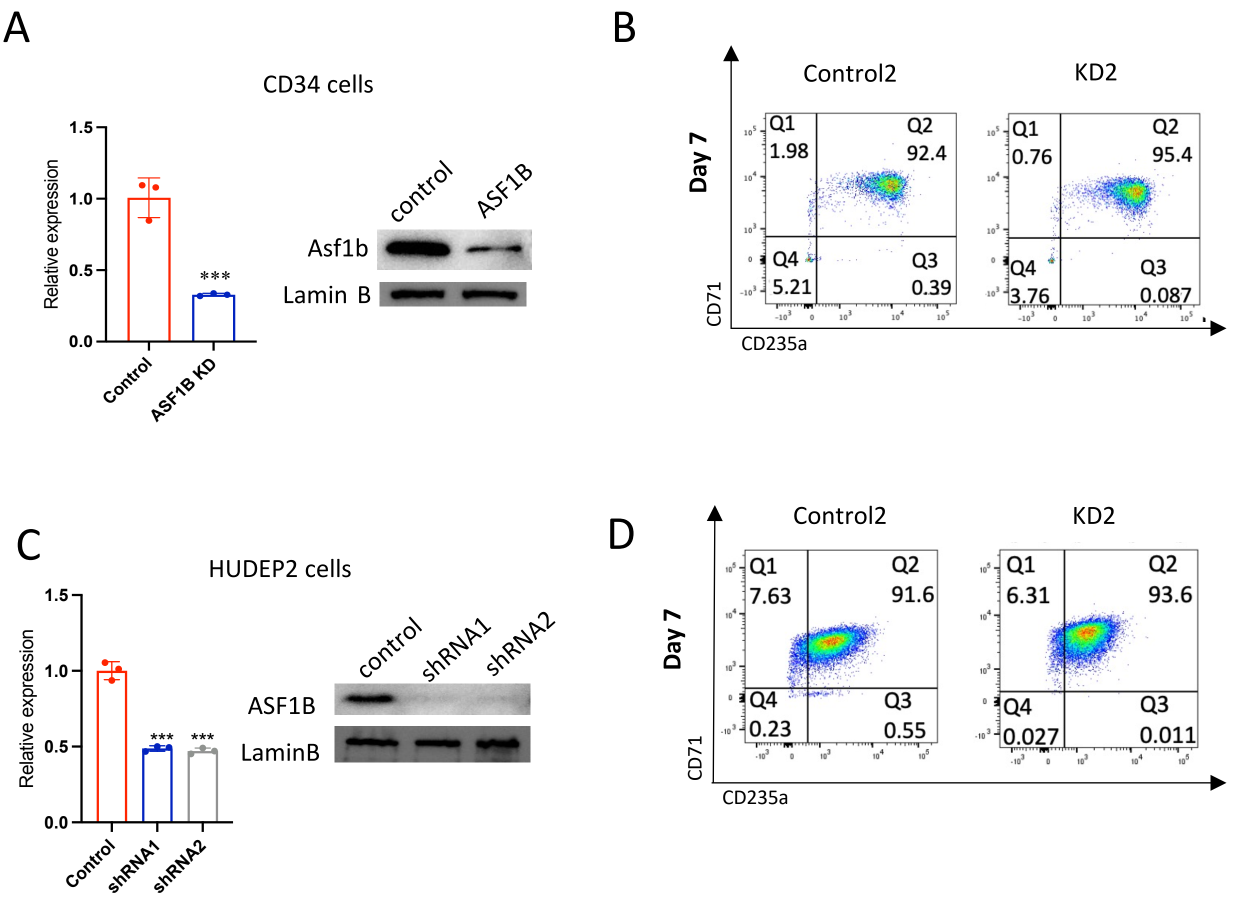
A
CD34 cells
Relative expression
1.5
1.0
0.5
0.0
***
Control
ASF1B KD
control
ASF1B
Asf1b
Lamin B
B
Control2
KD2
Day 7
Q1
1.98
Q2
92.4
Q4
5.21
Q3
0.39
Q1
0.76
Q2
95.4
Q4
3.76
Q3
0.087
CD71
CD235a
C
HUDEP2 cells
Relative expression
1.5
1.0
0.5
0.0
***
***
Control
shRNA1
shRNA2
control
shRNA1
shRNA2
ASF1B
LaminB
D
Control2
KD2
Day 7
Q1
7.63
Q2
91.6
Q4
0.23
Q3
0.55
Q1
6.31
Q2
93.6
Q4
0.027
Q3
0.011
CD71
CD235a

Figure S2. Loss of ASF1B impairs erythroid differentiation in human CD34+ cells and HUDEP2 cells.

A. RT-PCR and WB results identify knocking down (KD) of *ASF1B* gene in $CD34^+$ cells. **B**. Flow cytometry with CD235a and CD71 staining with *ASF1B* KD in $CD34^+$ cells and the control cells at differentiation day 7 (the second replication of Figure 1J). **C.** RT-PCR and Western blot results showing decrease of *ASF1B* expression and protein level in shRNA-mediated KD of HUDEP2 cells. **D**. Flow cytometry with CD235a and CD71 staining with *ASF1B* KD in HUDEP2 cells and the control cells at differentiation day 7 (the second replication of Figure 1K) . Error bars in panels **A** and **C** represent SD. N=3 biological replicates. * $P<0.05$, ** $P<0.01$, *** $P<0.001$ by two-tailed Student's t test.

Figure S3

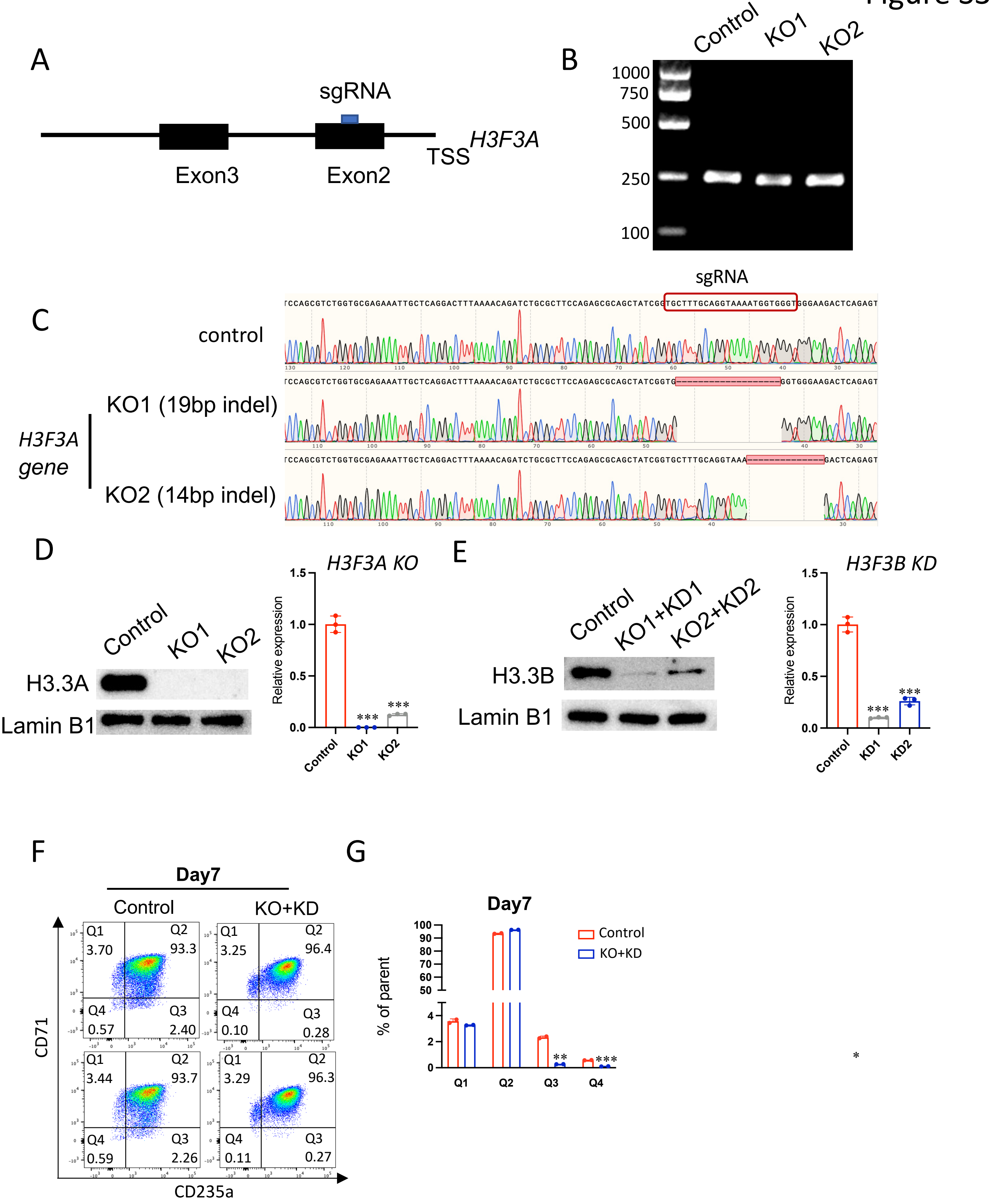
A
sgRNA
Exon3
Exon2
TSS
H3F3A
B
Control
KO1
KO2
1000
750
500
250
100
C
sgRNA
control
H3F3A gene
KO1 (19bp indel)
KO2 (14bp indel)
D
Control
KO1
KO2
H3.3A
Lamin B1
H3F3A KO
Relative expression
***
E
Control
KO1+KD1
KO2+KD2
H3.3B
Lamin B1
H3F3B KD
Relative expression
KD1
KD2
F
Day7
Control
KO+KD
Q1 3.70
Q2 93.3
Q4 0.57
Q3 2.40
Q1 3.25
Q2 96.4
Q4 0.10
Q3 0.28
Q1 3.44
Q2 93.7
Q4 0.59
Q3 2.26
Q1 3.29
Q2 96.3
Q4 0.11
Q3 0.27
CD71
CD235a
G
Day7
% of parent
Control
KO+KD
**
***
Q1
Q2
Q3
Q4

*

Figure S3. Generation of H3.3 deficient HUDEP2 cells.

A. Schematic diagram showing location of sgRNAs used to target *H3F3A* gene in HUDEP2 cells. **B-C**. PCR and sequencing validation of *H3F3A* gene deletions. The red dashed rectangle indicated the sgRNA sequence. **D-E.** Western blot and RT-PCR results showing changes of protein level and gene expression of H3.3A and H3.3B after *H3F3A* sgRNAs and *H3F3B* shRNAs treatment. **F**. Flow cytometry with CD235a and CD71 staining with H3.3 deficient HUDEP2 cells and the control cells at differentiation day 7. **G.** Quantification of cell numbers of Day 7 HUDEP2 cells in Panel **F**. Error bars in panels **E** and **G** represent SD. N=3 biological replicates. * $P<0.05$, ** $P<0.01$, *** $P<0.001$ by two-tailed Student's t test.

Figure S4

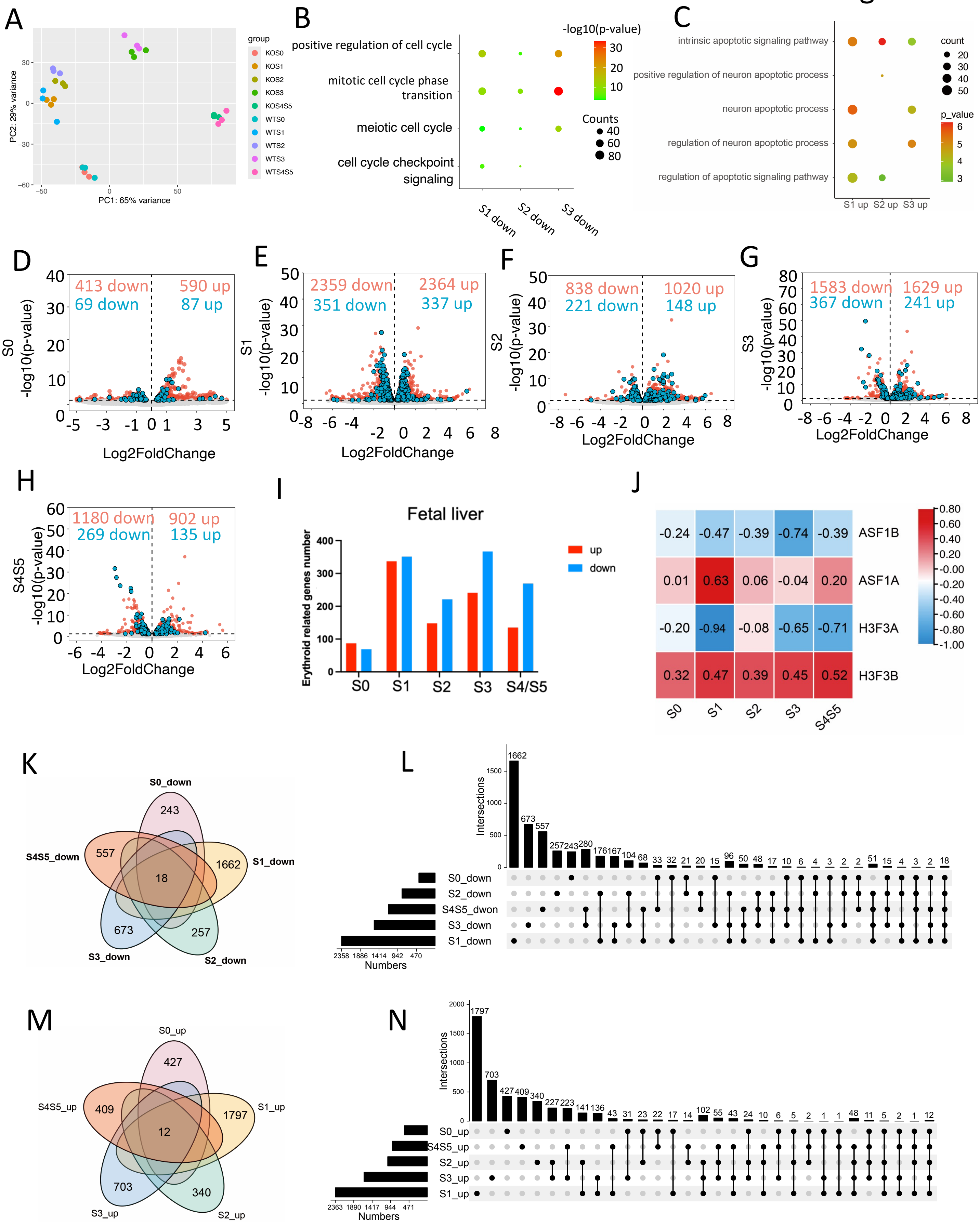
A
B
positive regulation of cell cycle
mitotic cell cycle phase transition
meiotic cell cycle
cell cycle checkpoint signaling
-log10(p-value)
Counts
S1 down
S2 down
S3 down
C
intrinsic apoptotic signaling pathway
positive regulation of neuron apoptotic process
neuron apoptotic process
regulation of neuron apoptotic process
regulation of apoptotic signaling pathway
count
p_value
S1 up
S2 up
S3 up
D
413 down
590 up
69 down
87 up
E
2359 down
2364 up
351 down
337 up
F
838 down
1020 up
221 down
148 up
G
1583 down
1629 up
367 down
241 up
H
1180 down
902 up
269 down
135 up
Log2FoldChange
I
Fetal liver
Erythroid related genes number
up
down
S0
S1
S2
S3
S4/S5
J
ASF1B
ASF1A
H3F3A
H3F3B
S4S5
K
S0_down
S1_down
S2_down
S3_down
S4S5_down
L
Intersections
S4S5_dwon
Numbers
M
S0_up
S1_up
S2_up
S3_up
S4S5_up
N

Figure S4. Absence of *Asf1b* caused significant alteration of the transcriptome during erythroid differentiation in mouse fetal liver cells.

A. PCA analysis of RNA-seq of S0-S5 population in *Asf1b* $^{-/-}$ and WT E14.5 liver cells. **B-C.** Gene ontology terms enriched among genes significantly down-regulated (B) and up-regulated (C) upon *Asf1b* KO in S1-S3 population cells. **D-H.** Volcano plot showing the number of total DEGs (red dots) and erythroid-related genes in DEGs (blue dots) between Asf1b$^{-/-}$ compared to WT E14.5 liver cells across S0-S5 population (p-value < 0.05). **I.** The number of up- and down-regulated erythroid-related genes shown in panel D-H. **J.** Log2 fold change of gene expression of *Asf1a*, *Asf1b*, *H3f3a* and *H3f3b* upon ASF1B KO revealed by RNA-seq in S0-S5 and E1-E4 population cells. **K-L.** Venn diagram (K) and UpSet plot (L) showing overlapped down-regulated genes upon *Asf1b* KO among E14.5 S0-S5 population cells. **M-N.** Venn diagram (M) and UpSet plot (N) showing overlapped up-regulated genes upon *Asf1b* KO among E14.5 S0-S5 population cells.

Figure S5

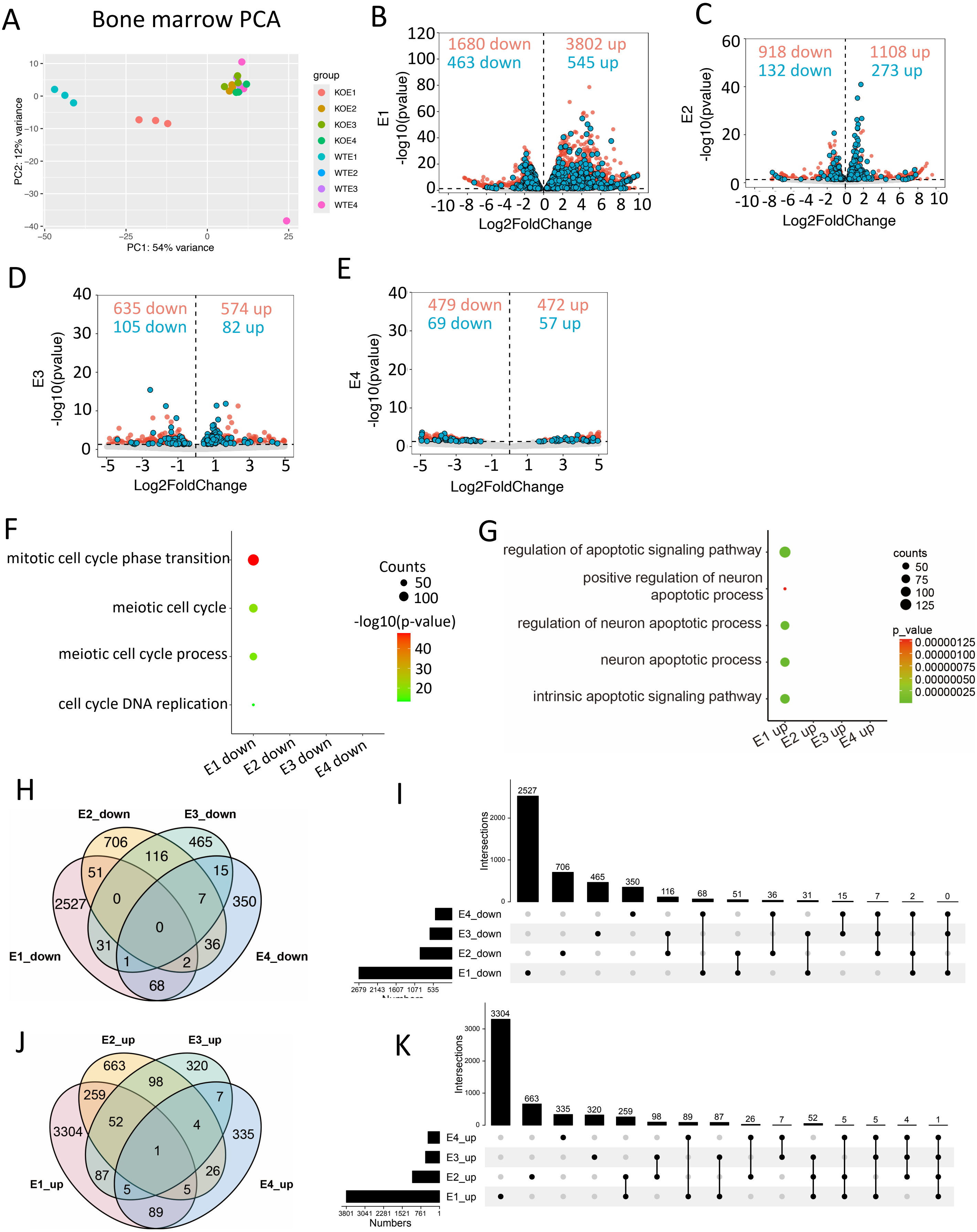
A
Bone marrow PCA
PC1: 54% variance
PC2: 12% variance
group
KOE1
KOE2
KOE3
KOE4
WTE1
WTE2
WTE3
WTE4
B
1680 down
463 down
3802 up
545 up
E1
-log10(pvalue)
Log2FoldChange
C
918 down
132 down
1108 up
273 up
E2
-log10(pvalue)
Log2FoldChange
D
635 down
105 down
574 up
82 up
E3
-log10(pvalue)
Log2FoldChange
E
479 down
69 down
472 up
57 up
E4
-log10(pvalue)
Log2FoldChange
F
mitotic cell cycle phase transition
meiotic cell cycle
meiotic cell cycle process
cell cycle DNA replication
Counts
50
100
-log10(p-value)
40
30
20
E1 down
E2 down
E3 down
E4 down
G
regulation of apoptotic signaling pathway
positive regulation of neuron apoptotic process
regulation of neuron apoptotic process
neuron apoptotic process
intrinsic apoptotic signaling pathway
counts
50
75
100
125
p_value
0.00000125
0.00000100
0.00000075
0.00000050
0.00000025
E1 up
E2 up
E3 up
E4 up
H
E1_down
E2_down
E3_down
E4_down
I
Intersections
E4_down
E3_down
E2_down
E1_down
J
E1_up
E2_up
E3_up
E4_up
K
Intersections
E4_up
E3_up
E2_up
E1_up
Numbers

Figure S5. Absence of *Asf1b* caused significant alteration of the transcriptome during erythroid differentiation in mouse bone marrow cells.

A. PCA analysis of RNA-seq of E1-E4 population in *Asf1b* $^{-/-}$ and WT bone marrow cells. **B-E.** Volcano plot showing the number of total DEGs (red dots) and erythroid-related genes in DEGs (blue dots) between Asf1b$^{-/-}$ compared to WT bone marrow cells across E1-E4 population (p-value < 0.05). **F-G.** Gene ontology terms enriched among genes significantly down-regulated (F) and up-regulated (G) upon *Asf1b* KO in bone marrow E1-E4 population cells. **H-I.** Venn diagram (H) and UpSet plot (I) showing overlapped down-regulated genes upon *Asf1b* KO among bone marrow E1-E4 population cells**.** **J-K.** Venn diagram (J) and UpSet plot (K) showing overlapped up-regulated genes upon *Asf1b* KO among bone marrow E1-E4 population cells.

Figure S6

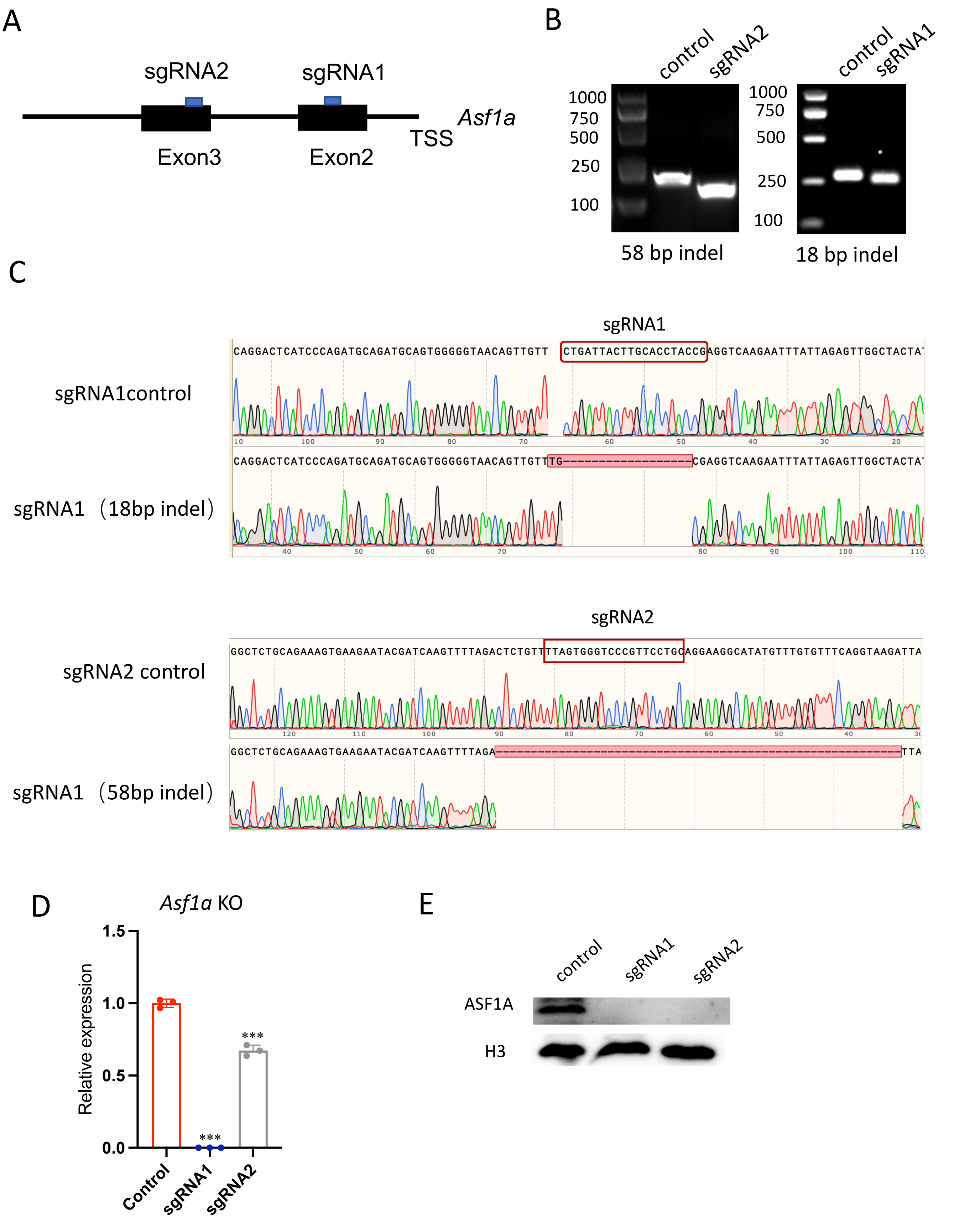

A
sgRNA2
sgRNA1
Asf1a
TSS
Exon3
Exon2
B
control
sgRNA2
control
sgRNA1
1000
750
500
250
100
58 bp indel
18 bp indel
C
sgRNA1
sgRNA1control
CAGGACTCATCCCAGATGCAGATGCAGTGGGGGTAACAGTTGTT CTGATTACTTGCACCTACCGAGGTCAAGAATTTATTAGAGTTGGCTACTA
sgRNA1 （18bp indel）
CAGGACTCATCCCAGATGCAGATGCAGTGGGGGTAACAGTTGTTTG
CGAGGTCAAGAATTTATTAGAGTTGGCTACTA
sgRNA2
sgRNA2 control
GGCTCTGCAGAAAGTGAAGAATACGATCAAGTTTTAGACTCTGTTTTAGTGGGTCCCGTTCCTGCAGGAAGGCATATGTTTGTGTTTCAGGTAAGATTA
sgRNA1 （58bp indel）
GGCTCTGCAGAAAGTGAAGAATACGATCAAGTTTTAGA
TTA
D
Asf1a KO
Relative expression
1.5
1.0
0.5
0.0
***
***
Control
sgRNA1
sgRNA2
E
control
sgRNA1
sgRNA2
ASF1A
H3

Figure S6. Generation of *Asf1a* KO MEL cells.

A. Schematic diagram showing location of sgRNAs used to target *Asf1a* gene in MEL cells. **B-C**. PCR and sequencing validation of *Asf1a* deletions. The red dashed rectangle indicated the sgRNA sequence. **D-E.** RT-PCR and Western blot results showing changes of *Asf1a* expression and protein level after sgRNAs treatment. Error bars represent SD. N=3 biological replicates. * $P<0.05$, ** $P<0.01$, *** $P<0.001$ by two-tailed Student's t test.

Figure S7

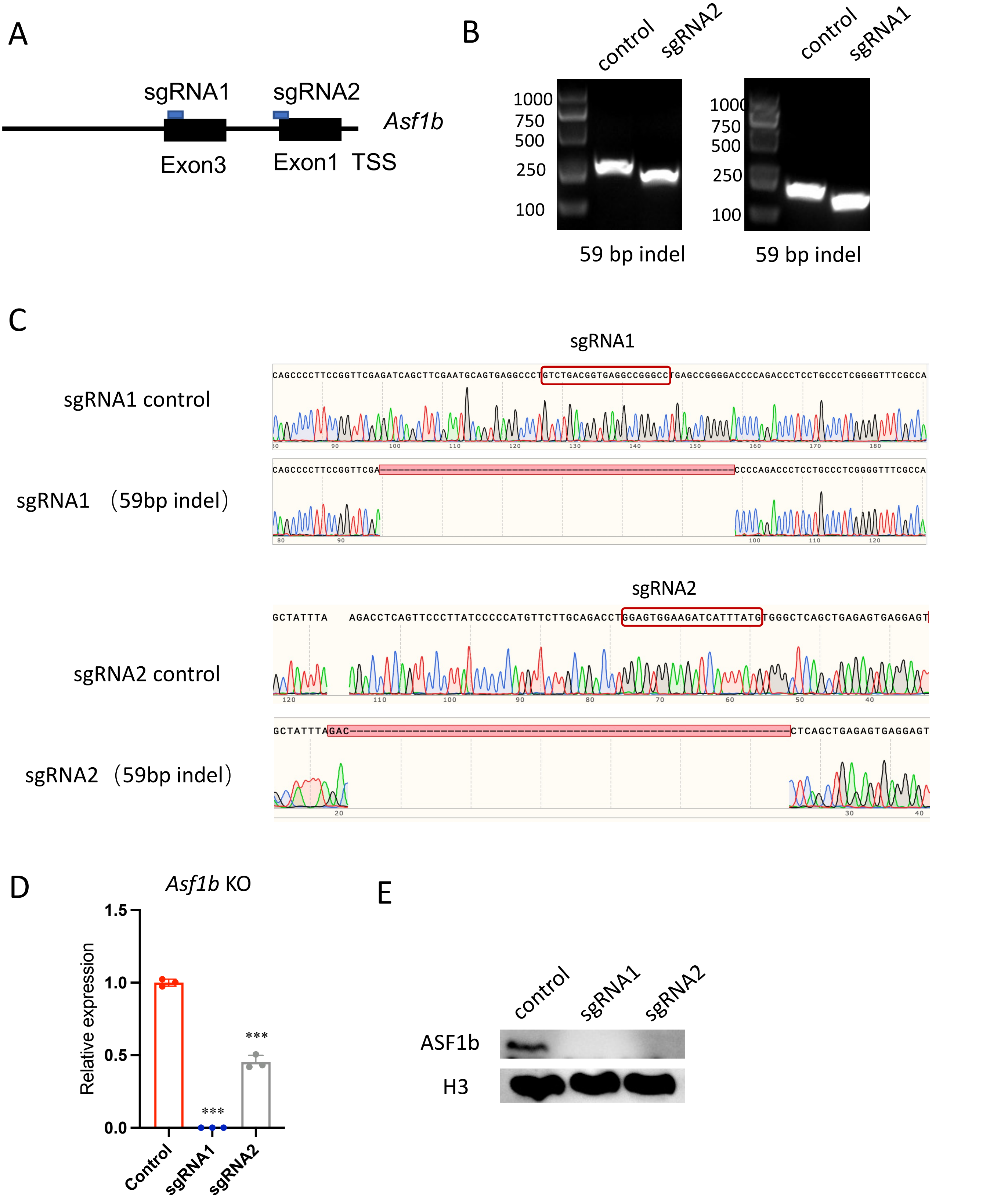
A
sgRNA1
sgRNA2
Asf1b
Exon3
Exon1 TSS
B
control
sgRNA2
control
sgRNA1
1000
750
500
250
100
1000
750
500
250
100
59 bp indel
59 bp indel
C
sgRNA1
sgRNA1 control
CAGCCCCTTCCGGTTCGAGATCAGCTTCGAATGCAGTGAGGCCCTGTCTGACGGTGAGGCCGGGCCTGAGCCGGGGACCCCAGACCCTCCTGCCCTCGGGGTTTCGCCA
sgRNA1 （59bp indel）
CAGCCCCTTCCGGTTCGA
CCCCAGACCCTCCTGCCCTCGGGGTTTCGCCA
sgRNA2
sgRNA2 control
GCTATTTA
AGACCTCAGTTCCCTTATCCCCCATGTTCTTGCAGACCTGGAGTGGAAGATCATTTATGTGGGCTCAGCTGAGAGTGAGGAGT
sgRNA2 （59bp indel）
GCTATTTAGAC
CTCAGCTGAGAGTGAGGAGT
D
Asf1b KO
Relative expression
1.5
1.0
0.5
0.0
***
***
Control
sgRNA1
sgRNA2
E
control
sgRNA1
sgRNA2
ASF1b
H3

Figure S7. Generation of *Asf1b* KO MEL cells.

A. Schematic diagram showing location of sgRNAs used to target *Asf1b* gene in MEL cells. **B-C**. PCR and sequencing validation of *Asf1b* deletions. The red dashed rectangle indicated the sgRNA sequence. **D-E.** RT-PCR and Western blot results showing changes of *Asf1b* expression and protein level after sgRNAs treatment. Error bars represent SD. N=3 biological replicates. * $P<0.05$, ** $P<0,01$, *** $P<0.001$ by two-tailed Student's t test.

Figure S8

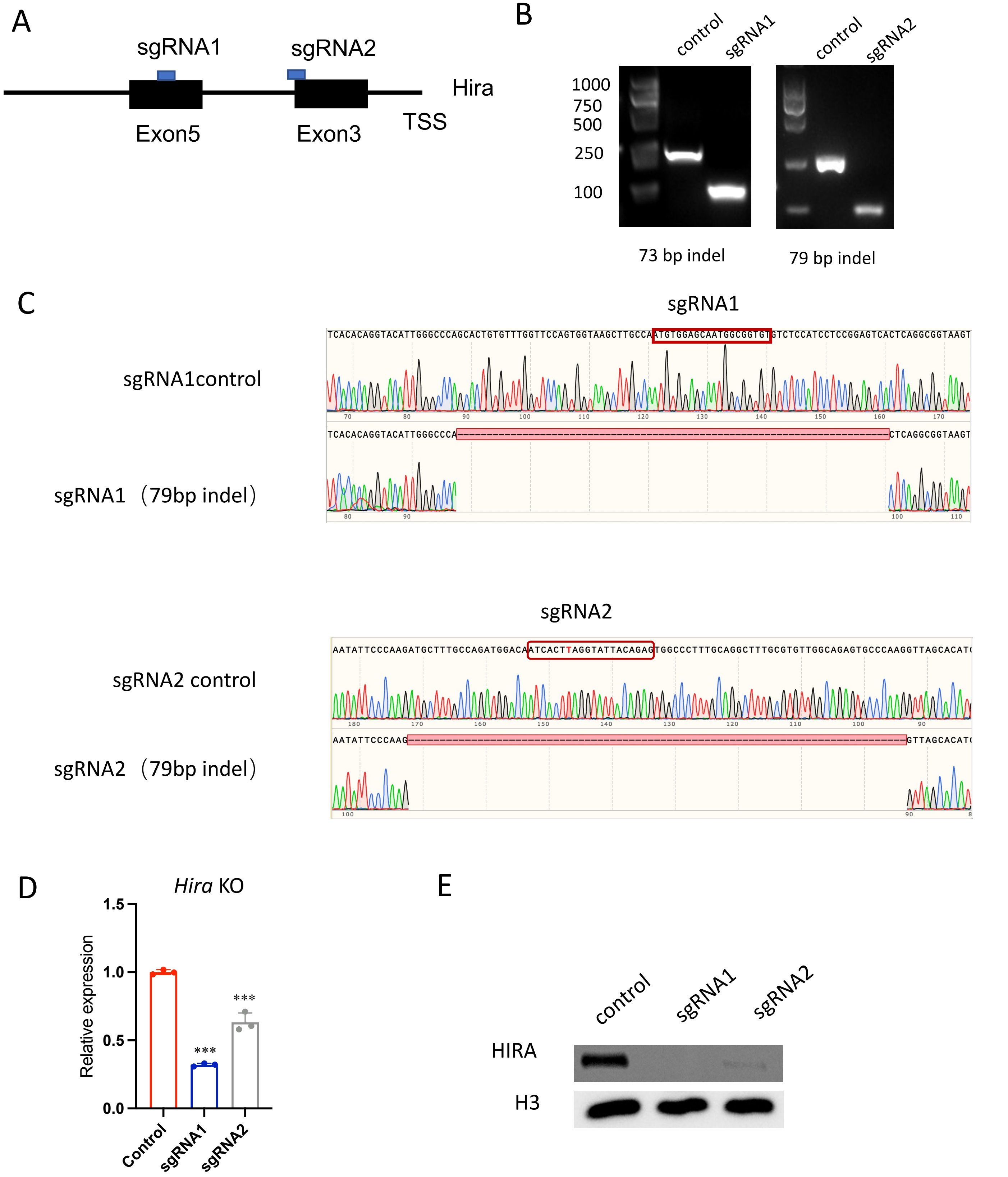
A
sgRNA1
sgRNA2
Hira
Exon5
Exon3
TSS
B
control
sgRNA1
control
sgRNA2
1000
750
500
250
100
73 bp indel
79 bp indel
C
sgRNA1
sgRNA1control
sgRNA1 （79bp indel）
sgRNA2
sgRNA2 control
sgRNA2 （79bp indel）
D
Hira KO
Relative expression
1.5
1.0
0.5
0.0
***
***
Control
sgRNA1
sgRNA2
E
control
sgRNA1
sgRNA2
HIRA
H3

Figure S8. Generation of *Hira* KO MEL cells.

A. Schematic diagram showing location of sgRNAs used to target *Hira* gene in MEL cells. **B-C**. PCR and sequencing validation of *Hira* deletions. The red dashed rectangle indicated the sgRNA sequence. **D-E.** RT-PCR and Western blot results showing changes of *Hira* expression and protein level after sgRNAs treatment. Error bars represent SD. N=3 biological replicates. * P<0.05, ** P<0,01, *** P<0.001 by two-tailed Student's t test.

Figure S9

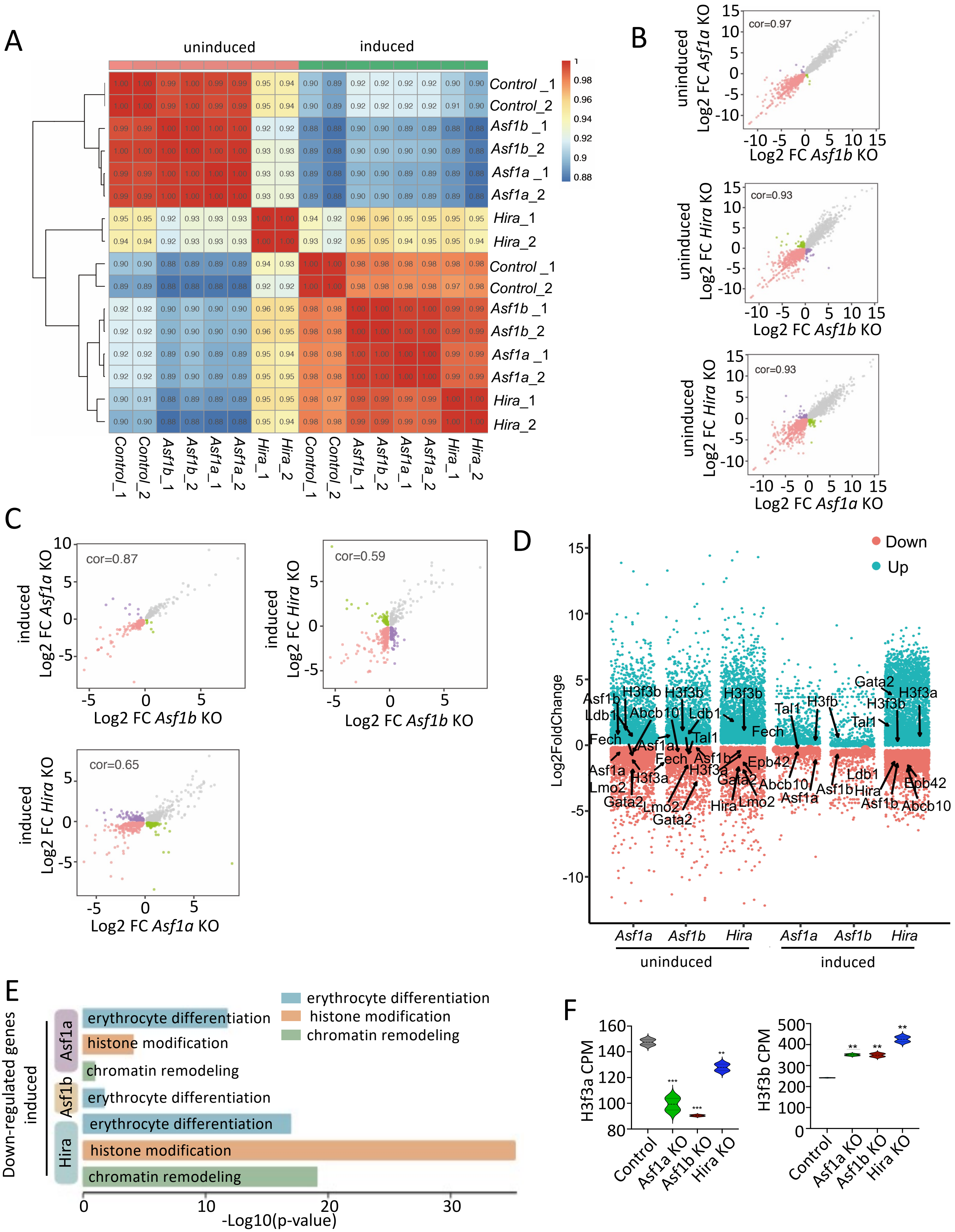
A
uninduced
induced
Control _1 1.00 1.00 0.99 1.00 0.99 0.99 0.95 0.94 0.90 0.89 0.92 0.92 0.92 0.92 0.90 0.90
Control_2 1.00 1.00 0.99 1.00 0.99 0.99 0.95 0.94 0.90 0.89 0.92 0.92 0.92 0.92 0.91 0.90
Asf1b _1 0.99 0.99 1.00 1.00 1.00 1.00 0.92 0.92 0.88 0.88 0.90 0.90 0.89 0.89 0.88 0.88
Asf1b_2 1.00 1.00 1.00 1.00 1.00 1.00 0.93 0.93 0.89 0.88 0.90 0.90 0.90 0.90 0.89 0.88
Asf1a _1 0.99 0.99 1.00 1.00 1.00 1.00 0.93 0.93 0.89 0.88 0.90 0.90 0.89 0.89 0.89 0.88
Asf1a_2 0.99 0.99 1.00 1.00 1.00 1.00 0.93 0.93 0.89 0.88 0.90 0.90 0.89 0.89 0.89 0.88
Hira_1 0.95 0.95 0.92 0.93 0.93 0.93 1.00 1.00 0.94 0.92 0.96 0.96 0.95 0.95 0.95 0.95
Hira_2 0.94 0.94 0.92 0.93 0.93 0.93 1.00 1.00 0.93 0.92 0.95 0.95 0.94 0.95 0.95 0.94
Control _1 0.90 0.90 0.88 0.89 0.89 0.89 0.94 0.93 1.00 1.00 0.98 0.98 0.98 0.98 0.98 0.98
Control_2 0.89 0.89 0.88 0.88 0.88 0.88 0.92 0.92 1.00 1.00 0.98 0.98 0.98 0.98 0.97 0.98
Asf1b _1 0.92 0.92 0.90 0.90 0.90 0.90 0.96 0.95 0.98 0.98 1.00 1.00 1.00 1.00 0.99 0.99
Asf1b_2 0.92 0.92 0.90 0.90 0.90 0.90 0.96 0.95 0.98 0.98 1.00 1.00 1.00 1.00 0.99 0.99
Asf1a _1 0.92 0.92 0.89 0.90 0.89 0.89 0.95 0.94 0.98 0.98 1.00 1.00 1.00 1.00 0.99 0.99
Asf1a_2 0.92 0.92 0.89 0.90 0.89 0.89 0.95 0.95 0.98 0.98 1.00 1.00 1.00 1.00 0.99 0.99
Hira_1 0.90 0.91 0.88 0.89 0.89 0.89 0.95 0.95 0.98 0.97 0.99 0.99 0.99 0.99 1.00 1.00
Hira_2 0.90 0.90 0.88 0.88 0.88 0.88 0.95 0.94 0.98 0.98 0.99 0.99 0.99 0.99 1.00 1.00
Control_1 Control_2 Asf1b_1 Asf1b_2 Asf1a_1 Asf1a_2 Hira_1 Hira_2 Control_1 Control_2 Asf1b_1 Asf1b_2 Asf1a_1 Asf1a_2 Hira_1 Hira_2
1 0.98 0.96 0.94 0.92 0.9 0.88
B
cor=0.97
uninduced Log2 FC Asf1a KO
Log2 FC Asf1b KO
cor=0.93
uninduced Log2 FC Hira KO
Log2 FC Asf1b KO
cor=0.93
uninduced Log2 FC Hira KO
Log2 FC Asf1a KO
C
cor=0.87
induced Log2 FC Asf1a KO
Log2 FC Asf1b KO
cor=0.59
induced Log2 FC Hira KO
Log2 FC Asf1b KO
cor=0.65
induced Log2 FC Hira KO
Log2 FC Asf1a KO
D
Down
Up
Log2FoldChange
Asf1b Ldb1 Fech H3f3b Abcb10 Asf1a Lmo2 Gata2 H3f3a Fech Ldb1 Tal1 Asf1b H3f3a Gata2 Lmo2 Gata2 Hira H3f3b Fech Epb42 Abcb10 Lmo2 Asf1a Tal1 H3fb Asf1b Hira Gata2 H3f3b Tal1 H3f3a Ldb1 Epb42 Asf1b Abcb10
Asf1a Asf1b Hira Asf1a Asf1b Hira
uninduced
induced
E
Down-regulated genes induced
Asf1a
Asf1b
Hira
erythrocyte differentiation
histone modification
chromatin remodeling
-Log10(p-value)
F
H3f3a CPM
H3f3b CPM
Control Asf1a KO Asf1b KO Hira KO
Control Asf1a KO Asf1b KO Hira KO
***
**

Figure S9. Absence of *Asf1b* resulted in a similar transcriptomic changes as absence of *Asf1a* in MEL cells.

A. Corrplot showing correlation of RNA-seq in control, *Asf1a*, *Asf1b* and *Hira* KO MEL cells in uninduced and induced conditions. **B-C.** Lfc-Lfc plot comparing two sets among dysregulated genes upon *Asf1b KO, Asf1a* KO and *Hira* KO in uninduced and induced MEL cells. **D.** Volcano plot showing the DEGs across 6 clusters in *Asf1a* KO, *Asf1b* KO and *Hira* KO compared to control MEL cells at uninduced and induced situation. **E.** Gene ontology terms enriched among genes significantly downregulated upon *Asf1a* KO, *Asf1b* KO and *Hira* KO in induced MEL cells. Blue represents erythrocyte differentiation pathways, yellow represents histone modification pathways, and green represents chromatin remodeling pathways. **F.** Counts Per Million (CPM) of RNA-seq data showing *H3f3a* and *H3f3b* gene expression level in WT and *Asf1a* KO, *Asf1b* KO, *Hira* KO MEL cells. n=3 biological replicates. * $P<0.05$, ** $P<0.01$, *** $P<0.001$ by two-tailed Student's t test.

Figure S10

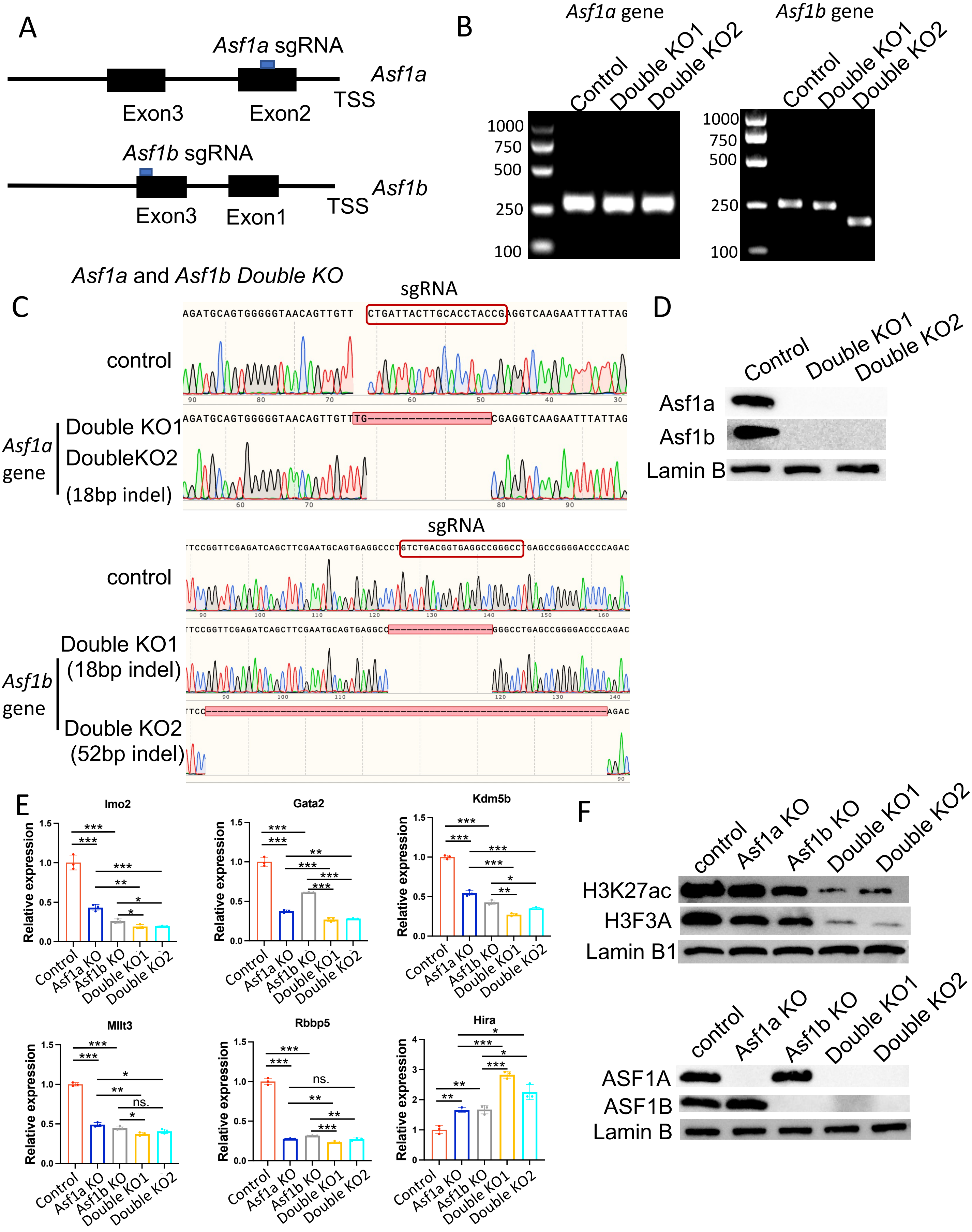

A
Asf1a sgRNA
Asf1a
Exon3
Exon2
TSS
Asf1b sgRNA
Asf1b
Exon3
Exon1
TSS
B
Asf1a gene
Asf1b gene
Control
Double KO1
Double KO2
1000
750
500
250
100
Asf1a and Asf1b Double KO
C
sgRNA
control
Asf1a gene
Double KO1
DoubleKO2
(18bp indel)
Asf1b gene
Double KO1
(18bp indel)
Double KO2
(52bp indel)
D
Control
Double KO1
Double KO2
Asf1a
Asf1b
Lamin B
E
Imo2
Gata2
Kdm5b
Mllt3
Rbbp5
Hira
Relative expression
Control
Asf1a KO
Asf1b KO
Double KO1
Double KO2
ns.
F
control
Asf1a KO
Asf1b KO
Double KO1
Double KO2
H3K27ac
H3F3A
Lamin B1
ASF1A
ASF1B
Lamin B

Figure S10. Generation of *Asf1a* and *Asf1b* double KO MEL cells.

A. Schematic diagram showing location of sgRNAs used to target *Asf1a* and *Asf1b* gene in MEL cells. **B-C.** PCR and sequencing validation of *Asf1a* and *Asf1b* deletions. The red dashed rectangle indicated the sgRNA sequences. **D.** Western blot results showing changes of Asf1a and Asf1b protein level after sgRNAs treatment. **E.** RT-PCR showing *Lmo2, Gata2, Kdm5b, Mllt3* , *Rbbp5* and *Hira* gene expression in *Asf1a* KO, *Asf1b* KO, Double KO and control MEL cells. Error bars represent SD. N=3 biological replicates. * $P<0.05$, ** $P<0.01$, *** $P<0.001$ by two-tailed Student's t test. **F.** Western blotting showing the protein level of H3.3,H3K27ac,ASF1A and ASF1B in Asf1a KO, Asf1b KO, Double KO and control MEL cells.

Figure S11

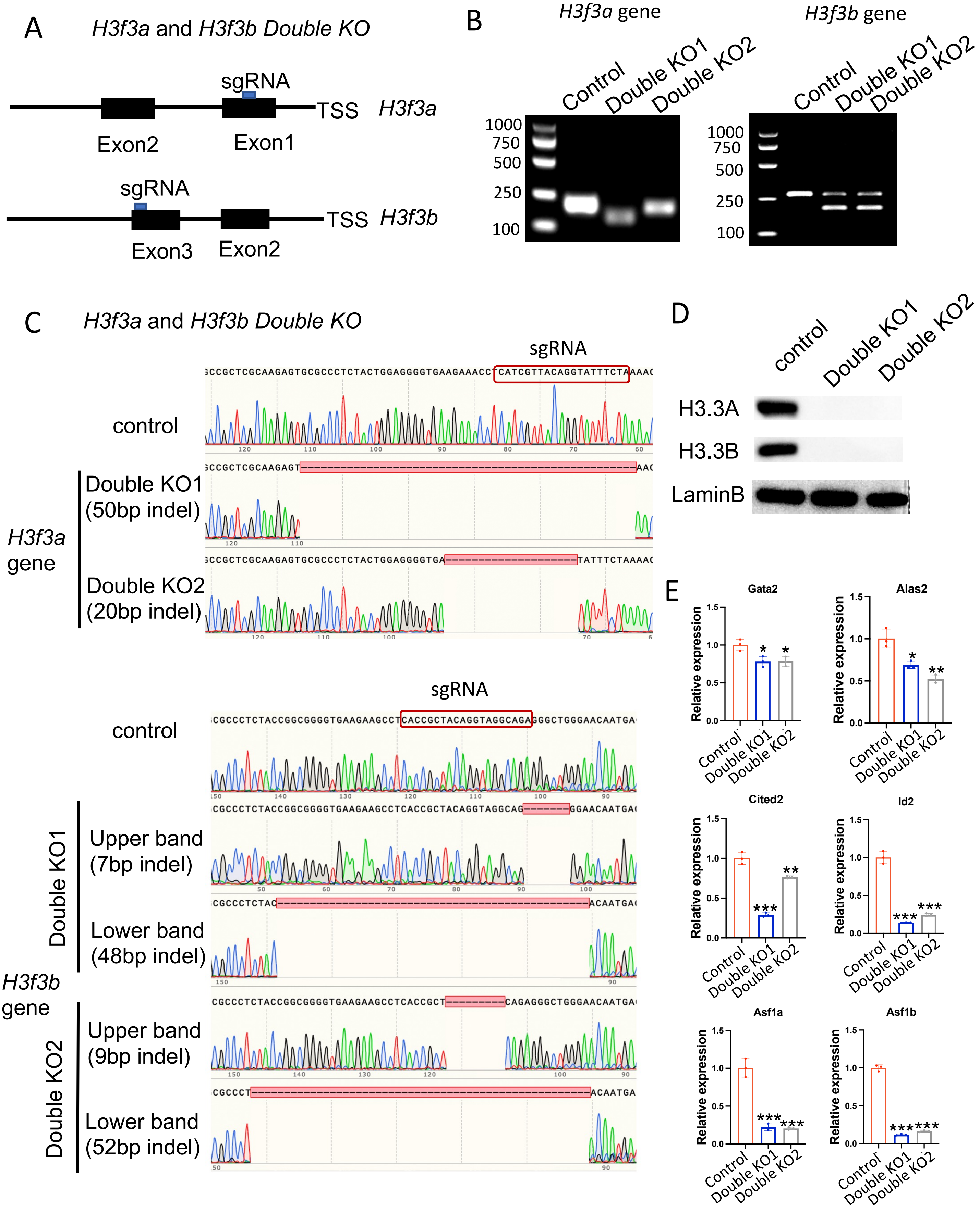
A
H3f3a and H3f3b Double KO
sgRNA
TSS
H3f3a
Exon2
Exon1
sgRNA
TSS
H3f3b
Exon3
Exon2
B
H3f3a gene
Control
Double KO1
Double KO2
1000
750
500
250
100
H3f3b gene
Control
Double KO1
Double KO2
1000
750
500
250
100
C
H3f3a and H3f3b Double KO
sgRNA
control
Double KO1
(50bp indel)
H3f3a
gene
Double KO2
(20bp indel)
sgRNA
control
Double KO1
Upper band
(7bp indel)
Lower band
(48bp indel)
H3f3b
gene
Double KO2
Upper band
(9bp indel)
Lower band
(52bp indel)
D
control
Double KO1
Double KO2
H3.3A
H3.3B
LaminB
E
Gata2
Alas2
Cited2
Id2
Asf1a
Asf1b
Relative expression
Control
Double KO1
Double KO2

Figure S11. Generation of double KO of *H3f3a* and *H3f3b* gene in MEL cells.

A. Schematic diagram showing location of sgRNAs used to target *H3f3a* and *H3f3b* gene in MEL cells. **B-C.** PCR and sequencing validation of *H3f3a* and *H3f3b* deletions in Double KO cells. The red dashed rectangle indicated the sgRNA sequence. **D.** Western blot results showing changes of protein level of H3.3 after sgRNAs treatment. **E.** RT-PCR showing *Gata2, Alas2, Cited2, Id2, Asf1a* and *Asf1b* gene expression in H3.3 Double KO and control MEL cells. Error bars represent SD. N=3 biological replicates. * $P<0.05$, ** $P<0,01$, *** $P<0.001$ by two-tailed Student's t test.

Figure S12

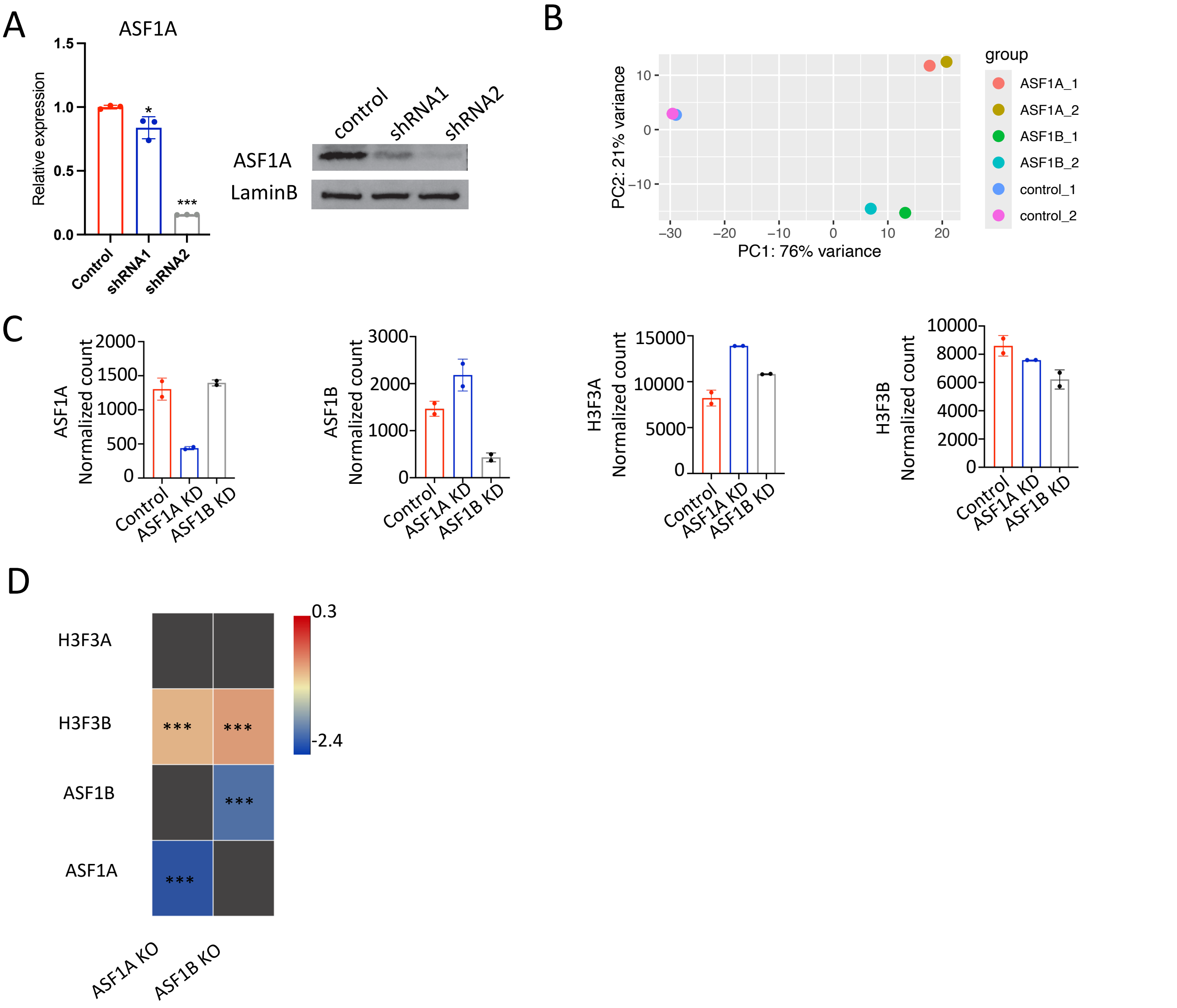
A
ASF1A
Relative expression
1.5
1.0
0.5
0.0
*
***
Control
shRNA1
shRNA2
control
shRNA1
shRNA2
ASF1A
LaminB
B
group
ASF1A_1
ASF1A_2
ASF1B_1
ASF1B_2
control_1
control_2
PC2: 21% variance
PC1: 76% variance
10
0
−10
−30
−20
−10
0
10
20
C
ASF1A
Normalized count
2000
1500
1000
500
0
Control
ASF1A KD
ASF1B KD
ASF1B
Normalized count
3000
2000
1000
0
Control
ASF1A KD
ASF1B KD
H3F3A
Normalized count
15000
10000
5000
0
Control
ASF1A KD
ASF1B KD
H3F3B
Normalized count
10000
8000
6000
4000
2000
0
Control
ASF1A KD
ASF1B KD
D
H3F3A
H3F3B
ASF1B
ASF1A
***
***
***
***
0.3
-2.4
ASF1A KO
ASF1B KO

Figure S12. Absence of *ASF1B* resulted in a similar transcriptomic changes as absence of *ASF1A* in human HUDEP2 cells.

A. RT-PCR and Western blot results showing decrease of *ASF1A* expression and protein level in shRNA-mediated KD HUDEP2 cells. Error bars represent SD. N=3 biological replicates. * $P<0.05$, ** $P<0,01$, *** $P<0.001$ by two-tailed Student's t test. **B.** PCA analysis of RNA-seq in *ASF1A* KD, *ASF1B* KD and control HUDEP2 cells. **C.** Normalized count showing gene expression of *ASF1A*, *ASF1B*, *H3F3A*, and *H3F3B* genes in control, *ASF1A* KD and *ASF1B* KD HUDEP2 cells. **D.** Heatmap shows log2 fold change of *ASF1A*, *ASF1B*, *H3F3A*, and *H3F3B* genes in panel C with P values superimposed. * $P<0.05$, ** $P<0,01$, *** $P<0.001$.

Figure S13

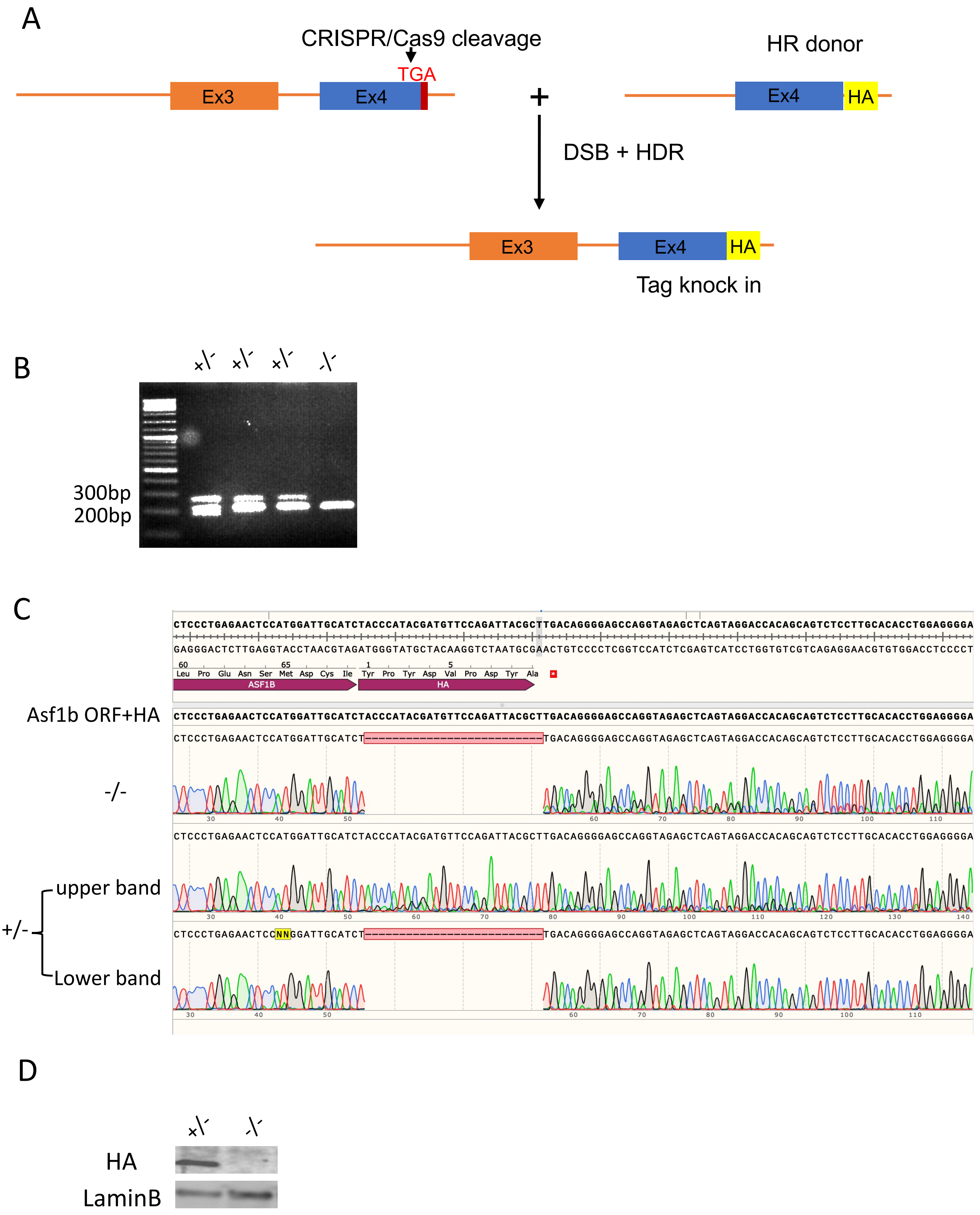

A
CRISPR/Cas9 cleavage
TGA
Ex3
Ex4
+
HR donor
Ex4
HA
DSB + HDR
Ex3
Ex4
HA
Tag knock in
B
+/- +/- +/- -/-
300bp
200bp
C
Leu Pro Glu Asn Ser Met Asp Cys Ile Tyr Pro Tyr Asp Val Pro Asp Tyr Ala
ASF1B
HA
Asf1b ORF+HA
-/-
upper band
+/-
Lower band
D
+/- -/-
HA
LaminB

Figure S13. Strategy for knocking in (KI) of HA-tag in *Asf1b* gene in mice. **A**. Diagram showing production of CRISPR/Cas9-mediated KI of HA tag in *Asf1b* gene. **B**. PCR showing fragment sizes for KI insertion (-/-, no insertion. +/- one copy of HA tag insertion). **C.** DNA sequencing track validation of HA tag insertion. **D.** Western blot showing insertion of HA tag.

Figure S14

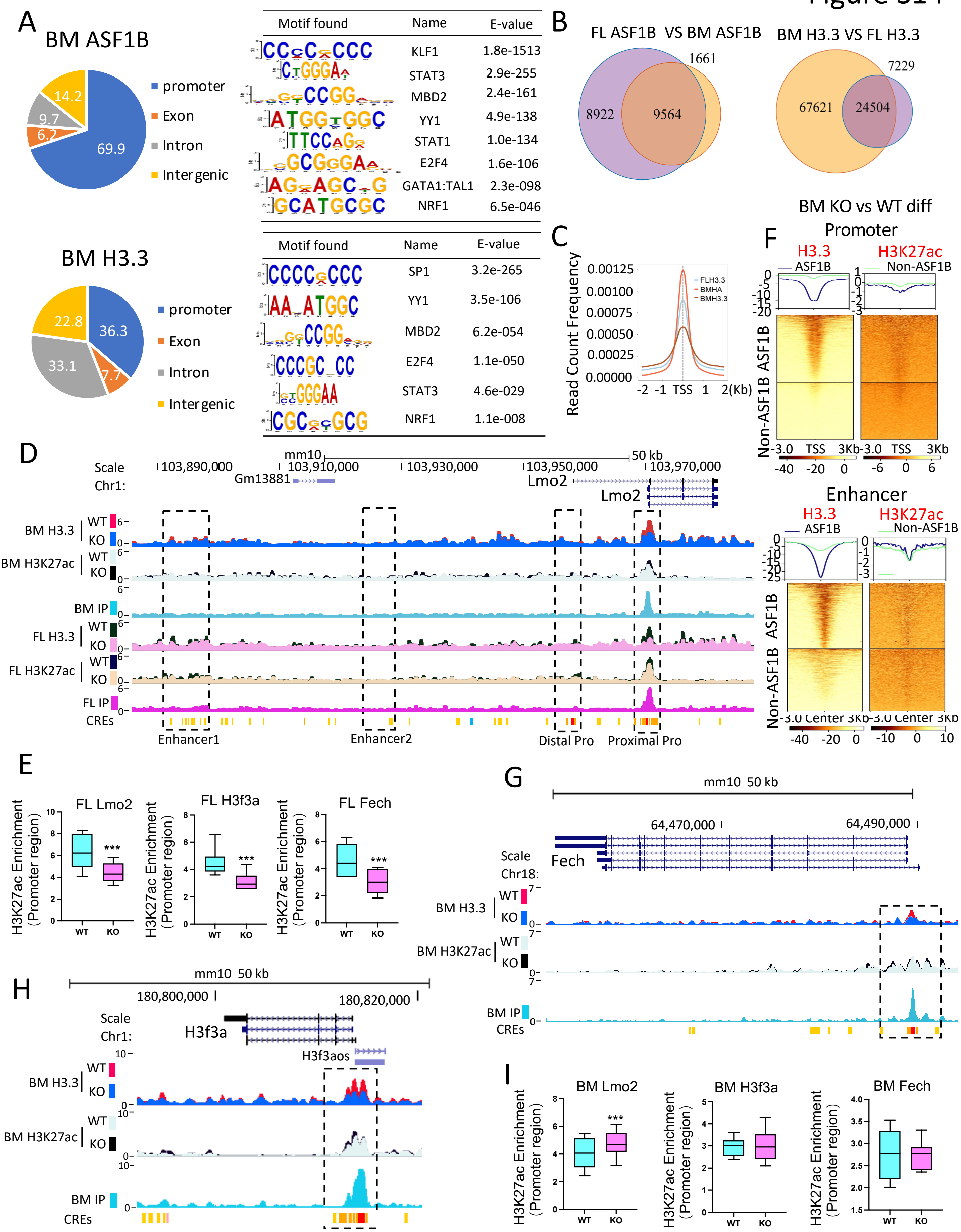

A
BM ASF1B
promoter
Exon
Intron
Intergenic
69.9
14.2
9.7
6.2
Motif found
Name
E-value
KLF1 1.8e-1513
STAT3 2.9e-255
MBD2 2.4e-161
YY1 4.9e-138
STAT1 1.0e-134
E2F4 1.6e-106
GATA1:TAL1 2.3e-098
NRF1 6.5e-046
BM H3.3
36.3
7.7
33.1
22.8
SP1 3.2e-265
YY1 3.5e-106
MBD2 6.2e-054
E2F4 1.1e-050
STAT3 4.6e-029
NRF1 1.1e-008
B
FL ASF1B VS BM ASF1B
8922
9564
1661
BM H3.3 VS FL H3.3
67621
24504
7229
C
Read Count Frequency
FLH3.3
BMHA
BMH3.3
-2 -1 TSS 1 2(Kb)
BM KO vs WT diff
Promoter
F
H3.3
H3K27ac
ASF1B
Non-ASF1B
Enhancer
-3.0 TSS 3Kb
-3.0 Center 3Kb
D
Scale
Chr1:
mm10
50 kb
103,890,000
103,910,000
103,930,000
103,950,000
103,970,000
Gm13881
Lmo2
BM H3.3
BM H3K27ac
BM IP
FL H3.3
FL H3K27ac
FL IP
WT
KO
CREs
Enhancer1
Enhancer2
Distal Pro
Proximal Pro
E
FL Lmo2
FL H3f3a
FL Fech
H3K27ac Enrichment (Promoter region)
WT
KO
***
G
mm10 50 kb
64,470,000
64,490,000
Scale
Chr18:
Fech
H
mm10 50 kb
180,800,000
180,820,000
H3f3a
H3f3aos
I
BM Lmo2
BM H3f3a
BM Fech

Figure S14. Loss of ASF1B decreased histone H3.3 in adult bone marrow cells.

A. Left, ChIP-seq with HA and H3.3 antibody showing the percentage of ASF1B peaks (BM ASF1B) and H3.3 enrichment (BM H3.3) in bone marrow (BM) cells at different genomic features. Right, Top motifs from *Meme* search of ASF1B and H3.3 enrichment sites from ChIP-seq data. **B.** Venn diagram of global overlapped sites of ASF1B ChIP-seq between E14.5 liver and bone marrow cells (FL ASF1B VS BM ASF1B), as well as H3.3 ChIP-seq between E14.5 liver and bone marrow cells (BM H3.3 VS FL H3.3). **C.** Peak density map for BM HA (ASF1B), FL H3.3 and BM H3.3 aligned at FL HA (ASF1B) peaks. **D.** Screen shot showing H3.3, H3K27ac and ASF1B ChIP-seq signals at promoter and enhancer of *Lmo2* gene locus. CREs indicated Cis-Regulatory Elements as promoter (red), enhancer (yellow) and CTCF sites (blue). **E.** Box plots showing the the quantification of H3K27ac ChIP-seq signals at the promoter regions (red) of the *Lmo2*, *H3f3a*, and *Fech* locus in E14.5 liver. **F.** Heatmap displaying differential ChIP-seq signals of H3.3 and H3K27ac when *Asf1b* KO and WT bone marrow cells in two categories (ASF1B-binding or non-ASF1B binding enhancer) at promoter (top) and enhancer (down). Top, each row represents a 3 kb window centered at Transcription Start Site (TSS). Down, each row represents a 3 kb window centered around the enhancer. **G-H.** Screen shot showing H3.3, H3K27ac and ASF1B ChIP-seq signals at promoter of *H3f3a* and *Fech* locus in bone marrow cells. CREs indicated Cis-Regulatory Elements as promoter (red), enhancer (yellow) and CTCF sites (blue). **I.** Box plots showing the the quantification of H3K27ac ChIP-seq signals at the promoter regions (red) of the *Lmo2*, *H3f3a*, and *Fech* locus in bone marrow cells. * $P<0.05$, ** $P<0,01$, *** $P<0.001$.

Figure S15

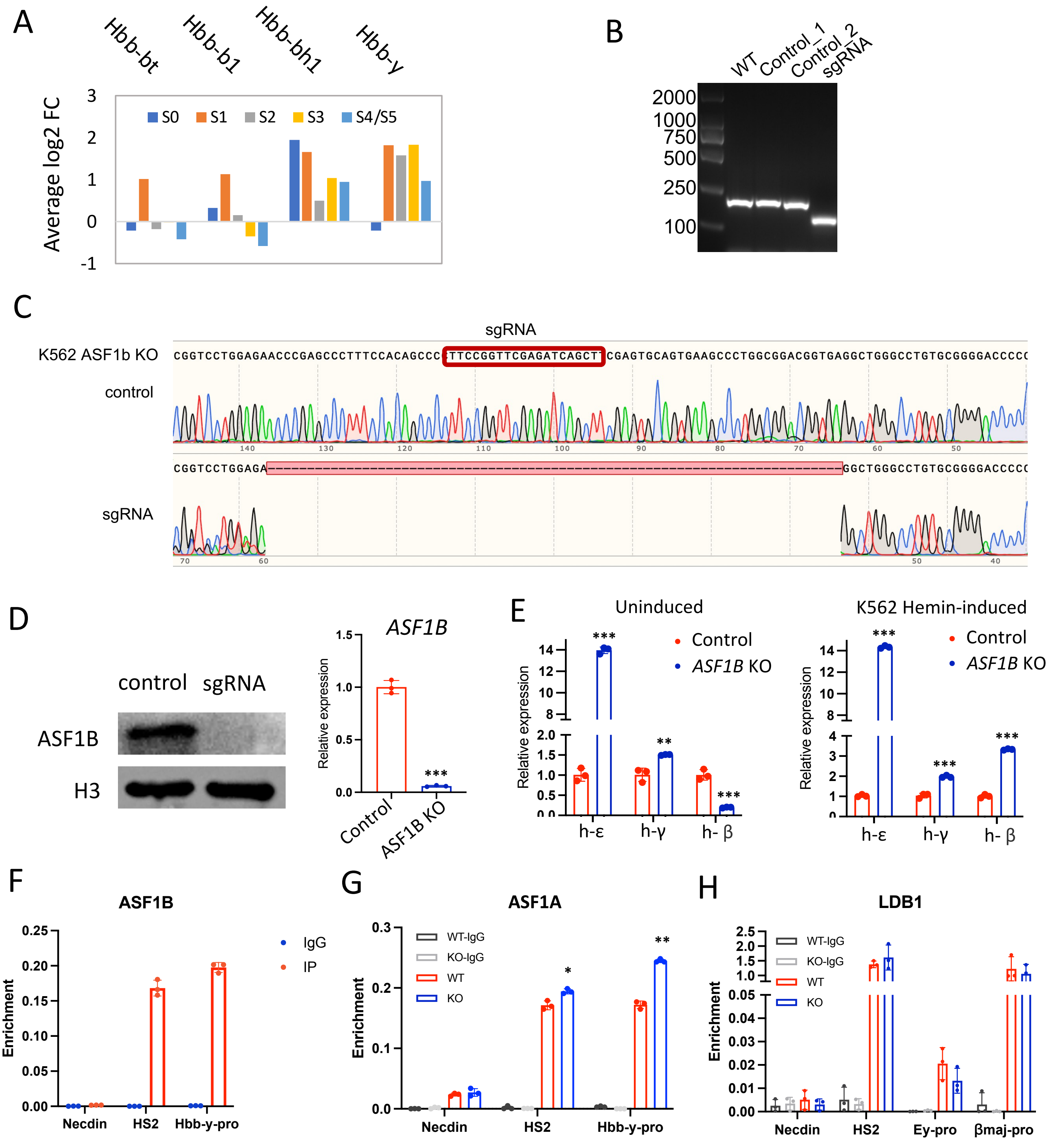

A
Hbb-bt
Hbb-b1
Hbb-bh1
Hbb-y
Average log2 FC
S0
S1
S2
S3
S4/S5
3
2
1
0
-1
B
WT
Control_1
Control_2
sgRNA
2000
1000
750
500
250
100
C
sgRNA
K562 ASF1b KO
CGGTCCTGGAGAACCCGAGCCCTTTCCACAGCCCTTTCCGGTTCGAGATCAGCTTCGAGTGCAGTGAAGCCCTGGCGGACGGTGAGGCTGGGCCTGTGCGGGGACCCCC
control
140
130
120
110
100
90
80
70
60
50
CGGTCCTGGAGA
GGCTGGGCCTGTGCGGGGACCCCC
sgRNA
70
60
50
40
D
control
sgRNA
ASF1B
H3
ASF1B
Relative expression
1.5
1.0
0.5
0.0
***
Control
ASF1B KO
E
Uninduced
K562 Hemin-induced
Control
ASF1B KO
Relative expression
14
12
10
8
2.0
1.5
1.0
0.5
0.0
4
3
2
1
0
***
**
h-ε
h-γ
h- β
F
ASF1B
Enrichment
0.25
0.20
0.15
0.10
0.05
0.00
IgG
IP
Necdin
HS2
Hbb-y-pro
G
ASF1A
Enrichment
0.3
0.2
0.1
0.0
WT-IgG
KO-IgG
WT
KO
*
**
Necdin
HS2
Hbb-y-pro
H
LDB1
Enrichment
2.5
2.0
1.5
1.0
0.05
0.04
0.03
0.02
0.01
0.00
WT-IgG
KO-IgG
WT
KO
Necdin
HS2
Ey-pro
βmaj-pro

Figure S15. ASF1B is associated with the repression of embryonic/fetal globin gene in mouse fetal liver and human K562 cells.

A. RNA-seq showing *Hbb-bh1*, *Hbb-y* and *Hbb-bs* globin gene expression changes in S3 and S4/5 populations of E14.5 liver cells upon KO of *Asf1b* gene. **B-D.** PCR, Sequencing validation, Western blot and RT-PCR results identify KO of ASF1B gene in K562 cells. The red dashed rectangle indicated the sgRNA sequence. **E**. RT-PCR showing ε-, γ-, and β-globin gene expression in *ASF1B* KO and control K562 cells in uninduced and Hemin-induced condition. **F.** ChIP-qPCR for ASF1B occupancy in the β-globin locus in E14.5 liver cells. **G.** ChIP-qPCR for ASF1A occupancy in the β-globin locus in *Asf1b* $^{-/-}$ and WT E14.5 liver cells. **H.** ChIP-qPCR for LDB1 occupancy in the β-globin locus in *Asf1b* $^{-/-}$ and WT E14.5 liver cells. Error bars in panels **D**, **E, F** and **G** represent SD. N=3 biological replicates. * $P<0.05$, ** $P<0.01$, *** $P<0.001$ by two-tailed Student's t test.

Figure S16

A

FL BRG1

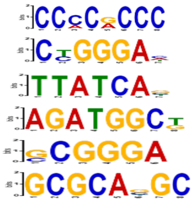

Motif found	Name	E-value
	KLF1	6.0e-186
	STAT1	6.1e-050
	Gata5	5.8e-024
	YY1	3.1e-010
	E2F4	1.1e-006
	NRF1	1.3e-006

B

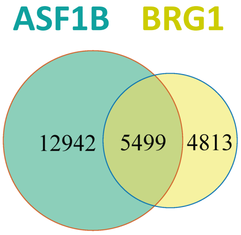

C

The genes co-occupies by Asf1b and BRG1

**Erythroid regulators**	**Cell proliferation**	**Cell Cycle**	**Cell Apoptosis**
Stat1	Chek2	Hes6	Bcl2l13
Lmo2	Pcna	Cux1	Tgm2
Stat5b	Mcm2	E2f1	Cul5
Ptbp3	Mcm5	E2f4	Mdm2
Klf13	Chaf1a	Gabpa	
Fech	Chaf1b		
Tal1			
Ldb1			
Med21			

D

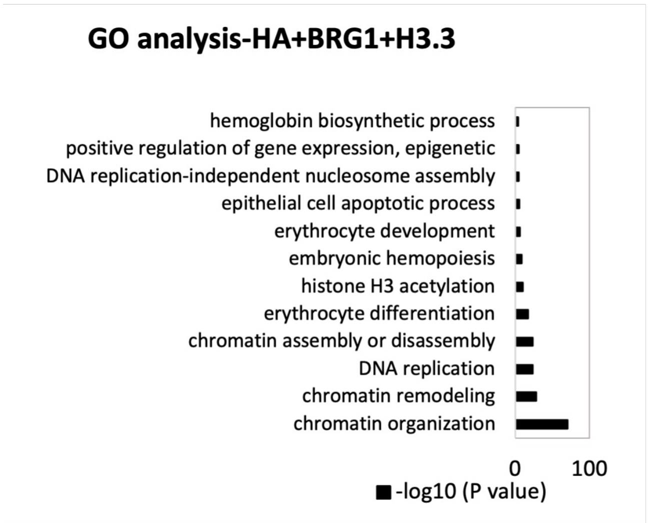

Figure S16. BRG1 occupied erythroid genes and was enriched in pathways regulated by ASF1B.

A. Top motifs from *Meme* search of BRG1 enrichment sites from ChIP-seq data in E14.5 liver cells. **B.** Venn diagram of global overlapped sites between ASF1B and BRG1 in E14.5 liver cells. **C.** Genes function as regulators of erythroid cell, cell proliferation, cell cycle and cell apoptosis whose promoters are enriched with ASF1B and BRG1 overlapped peaks. **D.** Gene ontology terms analysis of co-occupied peaks among ASF1B, BRG1 and H3.3 (HA+BRG1+H3.3).

Figure S17

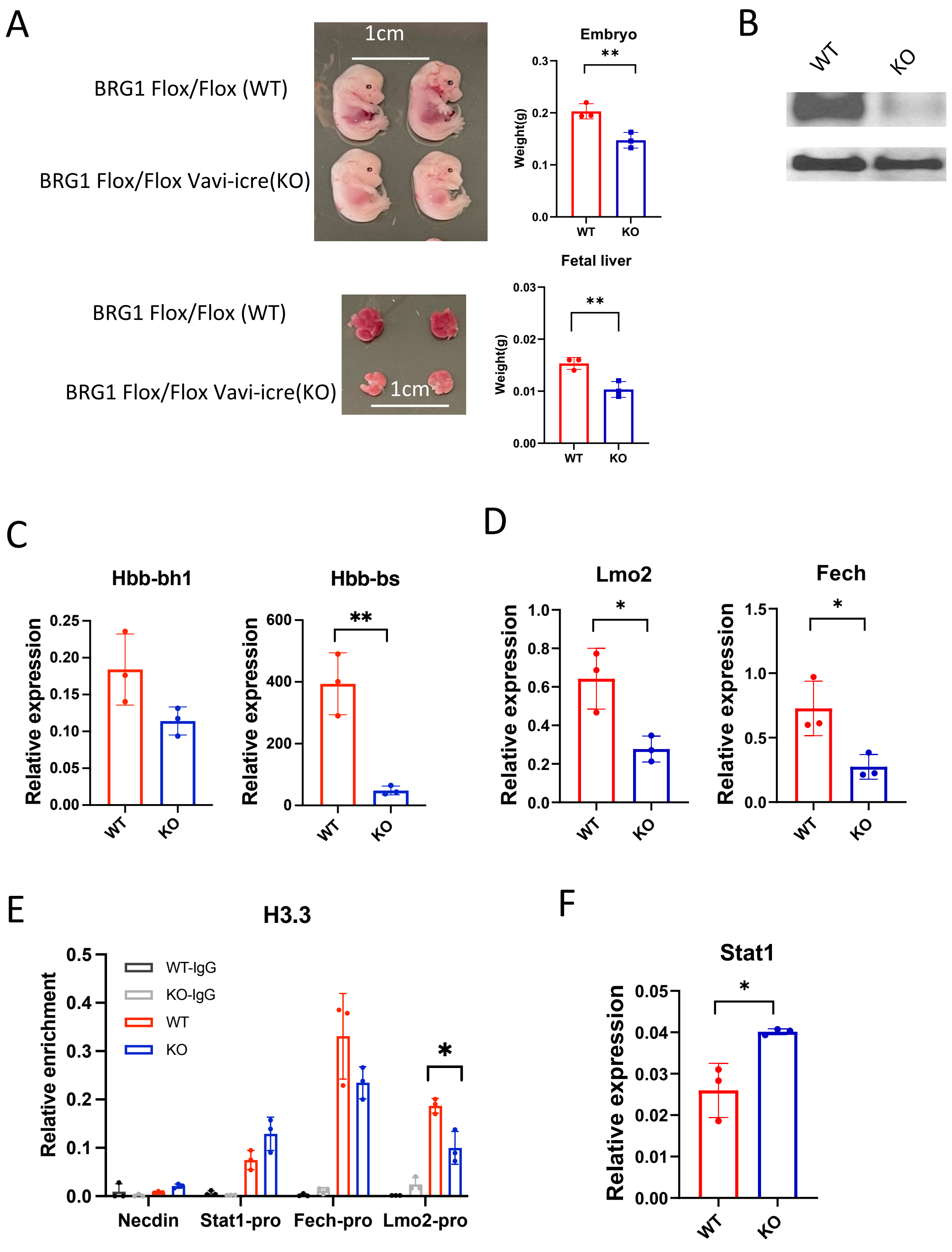
A
BRG1 Flox/Flox (WT)
BRG1 Flox/Flox Vavi-icre(KO)
1cm
Embryo
**
Weight(g)
0.3
0.2
0.1
0.0
WT
KO
BRG1 Flox/Flox (WT)
BRG1 Flox/Flox Vavi-icre(KO)
1cm
Fetal liver
**
Weight(g)
0.03
0.02
0.01
0.00
WT
KO
B
WT
KO
BRG1
LaminB
C
Hbb-bh1
Relative expression
0.25
0.20
0.15
0.10
0.05
0.00
WT
KO
Hbb-bs
**
Relative expression
600
400
200
0
WT
KO
D
Lmo2
*
Relative expression
1.0
0.8
0.6
0.4
0.2
0.0
WT
KO
Fech
*
Relative expression
1.5
1.0
0.5
0.0
WT
KO
E
H3.3
Relative enrichment
0.5
0.4
0.3
0.2
0.1
0.0
WT-IgG
KO-IgG
WT
KO
*
Necdin
Stat1-pro
Fech-pro
Lmo2-pro
F
Stat1
*
Relative expression
0.05
0.04
0.03
0.02
0.01
0.00
WT
KO

Figure S17. BRG1 is required for the enrichment of H3.3 at ASF1B-regulated genes.

A. E14.5 embryos and livers in KO (*Brg1* $^{-/-}$) mice are smaller in size with lower cell numbers compared to WT littermates. Scale bar, 1cm. **B.** Western Blotting showing the decreased protein level of BRG in Brg1 $^{-/-}$ mouse compared to WT littermates. **C.** RT-PCR showing expression of *Hbb-bh1* and *Hbb-bs* gene in *Brg1* $^{-/-}$ and WT E14.5 liver cells. **D.** RT-PCR showing expression of *lmo2* and *fech* gene in *Brg1* $^{-/-}$ and WT E14.5 liver cells. **E.** ChIP-qPCR for H3.3 enrichment at promoters of *stat1*, *fech* and *lmo2* gene in *Brg1* $^{-/-}$ and WT E14.5 liver cells. **F.** RT-PCR showing expression of *stat1* genes in *Brg1* $^{-/-}$ and WT E14.5 liver cells. Error bars in panels **A**, **C**, **D**, **E** and **F** represent SD. N=3 biological replicates. * $P<0.05$, ** $P<0.01$ by two-tailed Student's t test.
